# Investigation of human apoB48 metabolism using a new, integrated non‐steady‐state model of apoB48 and apoB100 kinetics

**DOI:** 10.1111/joim.12877

**Published:** 2019-03-12

**Authors:** E. Björnson, C. J. Packard, M. Adiels, L. Andersson, N. Matikainen, S. Söderlund, J. Kahri, C. Sihlbom, A. Thorsell, H. Zhou, M.‐R. Taskinen, J. Borén

**Affiliations:** ^1^ Department of Molecular and Clinical Medicine University of Gothenburg and Sahlgrenska University Hospital Gothenburg Sweden; ^2^ Institute of Cardiovascular and Medical Sciences University of Glasgow Glasgow UK; ^3^ Research Programs Unit, Diabetes and Obesity University of Helsinki Helsinki Finland; ^4^ Department of Internal Medicine Helsinki University Hospital Helsinki Finland; ^5^ Endocrinology, Abdominal Center Helsinki University Hospital Helsinki Finland; ^6^ Proteomics Facility University of Gothenburg Gothenburg Sweden; ^7^ Merck Research Laboratories Merck & Co. Inc. Kenilworth NJ USA

**Keywords:** apolipoprotein B48, kinetics, model, remnants, stable isotope

## Abstract

**Background:**

Triglyceride‐rich lipoproteins and their remnants have emerged as major risk factors for cardiovascular disease. New experimental approaches are required that permit simultaneous investigation of the dynamics of chylomicrons (CM) and apoB48 metabolism and of apoB100 in very low‐density lipoproteins (VLDL).

**Methods:**

Mass spectrometric techniques were used to determine the masses and tracer enrichments of apoB48 in the CM, VLDL_1_ and VLDL_2_ density intervals. An integrated non‐steady‐state multicompartmental model was constructed to describe the metabolism of apoB48‐ and apoB100‐containing lipoproteins following a fat‐rich meal, as well as during prolonged fasting.

**Results:**

The kinetic model described the metabolism of apoB48 in CM, VLDL_1_ and VLDL_2_. It predicted a low level of basal apoB48 secretion and, during fat absorption, an increment in apoB48 release into not only CM but also directly into VLDL_1_ and VLDL_2_. ApoB48 particles with a long residence time were present in VLDL, and in subjects with high plasma triglycerides, these lipoproteins contributed to apoB48 measured during fasting conditions. Basal apoB48 secretion was about 50 mg day^−1^, and the increment during absorption was about 230 mg day^−1^. The fractional catabolic rates for apoB48 in VLDL_1_ and VLDL_2_ were substantially lower than for apoB48 in CM.

**Discussion:**

This novel non‐steady‐state model integrates the metabolic properties of both apoB100 and apoB48 and the kinetics of triglyceride. The model is physiologically relevant and provides insight not only into apoB48 release in the basal and postabsorptive states but also into the contribution of the intestine to VLDL pool size and kinetics.

## Graphical abstract

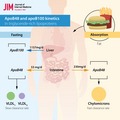

## Introduction

Recent genetic and epidemiological studies have provided evidence that plasma triglyceride‐rich lipoproteins (TRLs) play a causal role in cardiovascular disease, and this has prompted renewed interest in understanding better the metabolism of these lipoproteins and their potential contribution to atherogenesis 1, 2, 3. There are two major transporters of triglyceride in the circulation: apolipoprotein (apo) B100‐containing very low‐density lipoproteins (VLDL) which are released virtually constantly from the liver and apoB48‐containing chylomicrons which are secreted from the intestine in a wave during dietary fat absorption. TRLs in the circulation are acted on first by lipoprotein lipase to remove much of the core triglyceride, and the resulting remnants are cleared by cell‐surface receptors 4, or in the case of VLDL converted (in part) by further lipolysis to intermediate‐ and low‐density lipoproteins. These lipid metabolism pathways are quantitatively significant; during fasting conditions in healthy individuals, the liver releases about 20–70 g of VLDL triglyceride per day associated with about 1 g of apoB100, while the intestine absorbs and packages in the order of 50–200 g of triglyceride daily depending on the fat content of the diet 5, 6.

Conventionally, triglyceride‐rich lipoproteins are isolated by centrifugation and can be usefully divided into three fractions: chylomicrons (CM) with Sf (Svedberg flotation rate) >400, larger VLDL (VLDL_1_) with Sf 60‐400 and smaller VLDL (VLDL_2_) with Sf 20‐60. Newly secreted particles and their partially lipolysed remnants (which are believed to contribute particularly to the development of atherosclerotic plaque 7) can exist across the entire size and density range. It follows that development of a true picture of the physiology of TRLs requires investigation of the non‐steady‐state dynamics of chylomicron metabolism overlayered on the (near) steady‐state system of VLDL kinetics. To date, this has been difficult to achieve and it has been necessary to use analytical approaches that are arguably over‐simplistic and/or employ nonphysiological nutritional regimens 8, 9, 10, 11. For example, previous studies of apoB48 metabolism have utilized a continuous micro‐meal feeding pattern to generate a quasi‐steady state or used a single metabolic compartment to represent chylomicron kinetics following a test meal 8, 9, 12, 13, 14, 15, 16, 17, 18. The latter is closer to normal physiology than the former but since the model does not reflect the complexities of the system, it limits the ability to apply the findings to real‐life nutritional settings.

In the present investigation, we used multiple stable‐isotope tracers, advanced mass spectrometric techniques and developed a novel multicompartmental model to assess triglyceride, apoB48 and apoB100 kinetics throughout the lipolytic cascade. The development of advanced mass spectrometric techniques was critical for this study as they allowed the simultaneous measurement of concentrations and turnover kinetics of several apolipoproteins from a single digestion mixture with superior sensitivity and specificity 19, 20.

This integrated approach enabled deeper insight into the dynamics of triglyceride transport and its potential consequences for atherogenesis and will enable better understanding of the targets of new therapeutic agents. The model included elements that described successfully both the continuous flux of VLDL from the liver and the non‐steady‐state dynamics of chylomicron release in response to a fat meal. It was constructed using data from subjects with a range of fasting plasma triglyceride concentrations (from 0.74 to 5.7 mmol L^−1^) in order to ensure its wide applicability.

## Methods

### Subjects

Two groups, each comprising four male volunteers, were studied. They attended the metabolic clinic after an overnight fast from 8:00 pm the previous evening and were asked to refrain from strenuous exercise and alcohol for 3 days prior to each experimental phase. The protocol was approved by the Helsinki University Ethics Committee, and the volunteers gave written informed consent. The first group of four subjects were selected to have low, normal and elevated plasma triglyceride levels in order to develop a model that could be applied to the full range of triglyceride values. Each individual underwent two experimental protocols investigating apoB48 and apoB100 kinetics, one in which a fat‐rich test meal was administered and the other in the absence of a meal. The second group of four subjects had normal plasma triglyceride levels and were used to confirm the validity of the model (see Appendix S1). No subjects with the apoE2/2 phenotype were included in the study.

### Metabolic study protocol

To decipher the metabolism of postprandial lipid metabolism, we performed stable‐isotope studies both in the postprandial state and in the fasting state. Thus, each individual participated in two kinetic studies. On the evening of the first visit *(fasting state*), a blood sample was taken (Figure S1). At about 8.00 AM (study 0 h time‐point), deuterated leucine (5,5,5‐D3 Euriso‐Top, d3‐leucine) at a dose of 7 mg kg^−1^ body weight and deuterated glycerol (1,1,2,3,3‐D5 Euriso‐Top, d5‐glycerol) at an invariant dose of 500 mg (Isotec, Miamisburg, OH) were injected as a bolus. The blood sampling schedule was immediately before tracer injection and then at 2, 4, 6, 8, 10, 12, 15, 20, 30, and 45 min and 1, 2, 3, 4, 6, 8, 10 and 24 h for the measurement of d3‐leucine tracer concentrations in plasma. Samples taken before the injection of tracers and at 30, 45, 60, 75, 90, 120 and 150 min and 3, 4, 5, 6, 8, 10 and 24 h after were used for the measurement of d3‐leucine enrichment in apoB48 and apoB100 and d5‐glycerol enrichment in triglyceride in lipoprotein fractions. For the second visit (*postprandial state*), the study protocol was identical except that a mixed meal (68.5 g fat, 63 g carbohydrates and 40 g protein, total energy content 1032 kcal) was served 2 h after the infusions begin and consumed within 10 min.

### Lipoprotein preparation

Chylomicrons (CM; Sf > 400), VLDL_1_ (Sf 60‐400) and VLDL_2_ (Sf 20‐60) were isolated by preparative centrifugation as previously described 21. Plasma samples were overlayered with a gradient of salt solutions of decreasing density and centrifugation performed in three steps. Lipoprotein fractions were harvested from the top of the centrifuge tube and stored at −80 °C prior to processing.

### Quantification of apoB48 in plasma and lipoprotein fractions

Stable‐isotope‐labelled apoB48‐peptides (H‐LSQLQTYMI‐OH and H‐LSQLQTYMet(O)I‐OH) were purchased from JPT peptide Technologies (GmbH, Berlin, Germany). The stable isotopes ^13^C (x6) and ^15^N (x1) were incorporated into the isoleucine carboxyl terminal resulting in a mass shift of 7 Da. Purity for each peptide was >95%. The stable‐isotope‐labelled peptides were used as internal standards (ISTDs) for quantification of apoB48. The peptides were dissolved and diluted in 50 mmol L^−1^ ammonium bicarbonate to an ISTD master mix at concentrations of 0.025 pmol μL^−1^ for apoB48 and apoB48‐MetOx, respectively. Initially, calibration curves were prepared, in which the linearity of the mass spectrometric determination of the peptides was verified with the ISTD peptides spiked into representative pooled plasma samples. A total of ten plasma digests (20 μL) were prepared as described below with an equimolar mixture of the two apoB48‐peptides with varying concentrations ranging from 0.025 to 1.250 pmol. This corresponds to a concentration in the plasma of 1.25 to 50 fmol μL^−1^. Mass spectrometric analyses and evaluation were performed as described below. The mass spectrometry response was found to be linear within this concentration range for both peptides.

### Tryptic digestion of plasma, VLDL_1_, VLDL_2_ and chylomicron fractions

Plasma samples and fractions were thawed at room temperature and vortexed for 10 min. Twenty‐one microlitre of plasma was mixed with 189 μL 100 mmol L^−1^ TEAB (triethylammoinium bicarbonate), 147 μL 5% sodium deoxycholate and 44 μL of 50 mmol L^−1^ DTT (DL‐dithiothreitol, Sigma–Aldrich, Saint Louis, MO, USA). The samples were reduced for 20 min at 60 °C and then alkylated with 20 μL 200 mmol L^−1^ MMTS (S‐Methyl methanethiosulfonate, Sigma–Aldrich) for 20 min at room temperature. The reaction mixture was then diluted in 300 μL 100 mmol L^−1^ TEAB, 5 μL apoB48‐peptide internal standard (0.125 pmol) was added, and the mixture was digested overnight with 70 μL (0.2 mg mL^−1^) trypsin (Pierce) at 37 °C. A second aliquot of trypsin 70 μL (0.2 mg mL^−1^) was added, and digestion was continued for 3 h at 37 °C. Twenty‐one microlitre of VLDL fractions was diluted five times in 100 mmol L^−1^ TEAB, and 60 μL of the chylomicron fraction was diluted in 140 mmol L^−1^ TEAB (40 μL). Seventy microlitre 5% sodium deoxycholate and 21 μL 50 mmol L^−1^ DTT were added, and samples were reduced for 20 min at 60 °C and then alkylated with 10.5 μL 200 mmol L^−1^ MMTS for 20 min at room temperature. The reaction mixture was then diluted in 147 μL 100 mmol L^−1^ TEAB, 5 μL apoB48‐peptide internal standard (0.125 pmol) was added, and the mixture was digested overnight with 2 μL (0.1 mg mL^−1^) trypsin at 37 °C. A second aliquot of trypsin 2 μL (0.1 mg mL^−1^) was added, and digestion was continued for 3 h at 37 °C. The trypsin was inactivated by heating the samples to 90 °C for 10 min before immuno‐enrichment.

### Immuno‐enrichment of apoB48

Since apoB48 is a low‐abundance protein, we utilized antipeptide antibodies to enrich apoB48 from digested samples, namely the stable‐isotope‐labelled standards and capture by antipeptide antibody approach 22. Antibodies for apoB48 were coupled to magnetic beads (Dynabeads^®^ Protein A, Life Technologies, Oslo, Norway) according to manufacturer's instructions 20. After coupling, the bead‐antibody complex was blocked in Roti^®^ block (Carl Roth GmbH, Karlsruhe, Germany), for 30 min at room temperature. Antibody (10 μg) was used to couple 42 μL of protein A beads. Coupled beads were resuspended in the same volume 0.1% PBS‐Tween‐20 solution. For enrichment of apoB48 peptide in plasma, 100 μL 0.2% PBS‐Tween‐20 with 10X protease inhibitor (cOmplete Tablets, Mini, EDTA‐free, Roche Diagnostics GmbH, Mannheim, Germany), and antibody‐coupled beads (42 μL beads coupled to 10 μg antibody) were added to 880 μL digested plasma. For enrichment of apoB48 peptide in VLDL and chylomicron fractions, 37 μL 0.2% PBS‐Tween‐20 with 10X protease inhibitor, (cOmplete Tablets, Mini, EDTA‐free, Roche Diagnostics GmbH, Mannheim, Germany) and antibody‐coupled beads (21 μL beads coupled to 5 μg antibody) were added to 330 μL digested fraction. The mixture was incubated at 4 °C overnight with rotation. The next day, beads were washed twice with 0.6 mL 0.02% PBS‐Tween‐20 solution, once with 0.6 mL PBS and twice with 0.6 mL 100 mmol L^−1^ TEAB. Peptides were eluted with 50 μL elution buffer (water solution containing 1% formic acid) at 37 °C for 15 min with gentle rocking. The eluted samples were desalted on a SOLAμ HRP 96‐well plate (Thermo Scientific) using a positive pressure manifold (Biotage) and spun in a Speed‐Vac with low heat to dryness. For LC/MS analysis, the samples were resuspended in 15 μL solution containing 3% acetonitrile and 3% formic acid.

### Determination of leucine enrichment in apoB100 and of glycerol enrichment in triglycerides

ApoB100 isolated from VLDL_1_ and VLDL_2_ was hydrolysed, derivatized and subjected to gas chromatography mass spectrometry (GC/MS) to measure tracer leucine enrichment 5, 23. Triglycerides were isolated from lipoproteins and the tracer glycerol enrichment determined as previously described 5.

### Biochemical analyses

Fasting plasma glucose, triglycerides, HDL cholesterol, LDL cholesterol and plasma liver enzymes were determined by automated enzymatic methods using the Konelab 60i analyser (Thermo Fisher Scientific, Finland). Plasma levels of apoCIII were measured immunoturbidometrically (Kamiya Biomedical Company, Seattle, WA, USA). Fasting and postprandial apolipoprotein (apo) B48 and apoB100 levels in total plasma were measured by ELISA (Shibayagi, Shibukawa, Japan). Plasma non‐esterified fatty acids (NEFA) were analysed with an automated enzymatic colorimetric method (Wako Chemicals, Neuss, Germany). Postheparin LPL and HL activities were measured as described 24.

## Results

Characteristics of the four subjects used to develop the model in terms of age, sex, body weight and plasma lipid indices are given in Table 1. Body weights and BMI were similar, while plasma triglyceride levels (by design) varied from low normal (0.74 mmol L^−1^) to high (5.7 mmol L^−1^). Each person underwent two studies, that is with and without administration of a fat‐rich test meal and the time between the two experimental phases varied from 2 to 8 months. The values in Table 1 were obtained during the study phase when the meal was given.

**Table 1:** Characteristics of the four subjects. Subjects had similar anthropometric measurements; BMI, body weight and waist but showed variation in lipid‐related variables; plasma triglycerides, apoC‐III, apoB, LDL‐C and HDL‐C. All variables except LPL/HL activity are measured during the postprandial test day

	Subject 1	Subject 2	Subject 3	Subject 4
Age (y)	59	43	64	56
Body weight (kg)	92.5	89.1	93.1	98.5
BMI (kg m^−2^)	30.1	29.8	31.8	30.6
Waist (cm)	104	98	113	100
Triglycerides (mmol L^−1^)	0.74	1.1	2.1	5.7
ApoC‐III (mg dL^−1^)	9.9	11.7	15.6	22.8
ApoB (mg dL^−1^)	69	96	115	125
Total chol (mmol L^−1^)	4.7	5.1	5.4	6.9
LDL chol (mmol L^−1^)	2.7	3.4	3.1	3.8
HDL chol (mmol L^−1^)	1.7	1.3	1.3	0.85
FFA (μmol L^−1^)	450	372	351	260
LPL activity (mU mL^−1^)	199	130	129	114
HL activity (mU mL^−1^)	232	254	178	210

**Figure 1:**
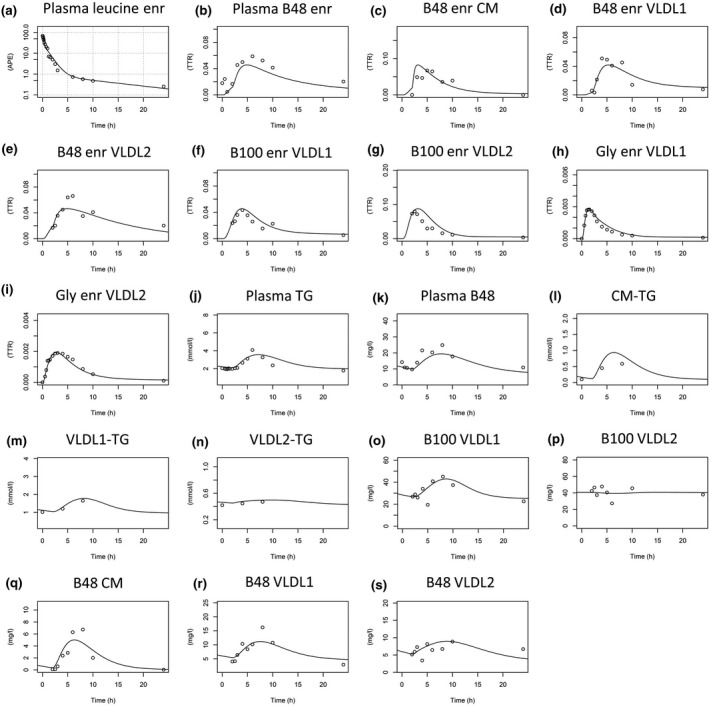
Model fits to experimental data for subject 3 – a representative subject. All tracer‐to‐tracee ratio data (for leucine and glycerol enrichment) in the different fractions are depicted in sub figures a–i, followed by concentration data depicted in subfigures j–s. For model fits to all subjects, see Appendix S1. Plasma leucine enr (enrichment) is plotted in semilogarithmic scale. APE, atom percent excess; TTR, tracer‐to‐tracee ratio.

Data from which to develop the multicompartmental model were extensive, and a typical set (for subject 3) is provided in Fig. 1. During the study phase, when a test meal was given, it comprised, in addition to plasma free leucine enrichment (panel A), (i) the change in concentration of triglyceride in plasma, CM, VLDL_1_ and VLDL_2_ (panels J, L, M, N); (ii) the change in concentration of apoB48 in plasma, CM, VLDL_1_ and VLDL_2_ (panels K, Q, R, S); (iii) the change in concentration of apoB100 in VLDL_1_ and VLDL_2_ (panels O, P); (iv) the enrichment of deuterated glycerol in triglyceride in VLDL_1_ and VLDL_2_ (panels H, I); (v) the enrichment of deuterated leucine in apoB48 in plasma, CM, VLDL_1_ and VLDL_2_ (panels B, C, D, E); and (vi) the enrichment of deuterated leucine in apoB100 in VLDL_1_ and VLDL_2_ (panels F, G). The model was fitted to all components (pools and enrichments) simultaneously, and as seen in Fig. 1, there was good agreement between the observed data (circles) and the simulated curves (lines).

**Figure 2:**
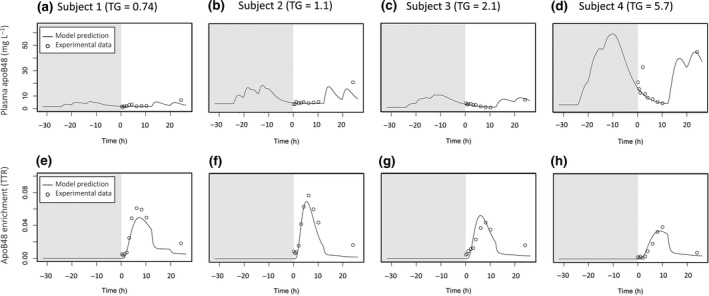
Model fits to plasma apoB48 and plasma apoB48 enrichment during fasting conditions for the four subjects. Since data were not available in the CM, VLDL_1_ and VLDL_2_ fraction, the model was fitted to only the total plasma measurements. Total plasma apoB48 concentration is shown in the top row (a–d) for each subject, and total plasma apoB48 enrichment is shown in the bottom row (e–h) for each subject. Modelling of the previous day is indicated with grey background.

Change in plasma apoB48 concentrations and apoB48 enrichment across the experimental observation period for the study phase when the subjects were not given a test meal are given in Fig. 2. Note the observations start at time 0 h and the simulated prior day was incorporated as described below. These data were also submitted to modelling in a separate fitting process to generate production and clearance rates and change in concentration for apoB48 in fasting conditions.

### Overall model structure

**Figure 3:**
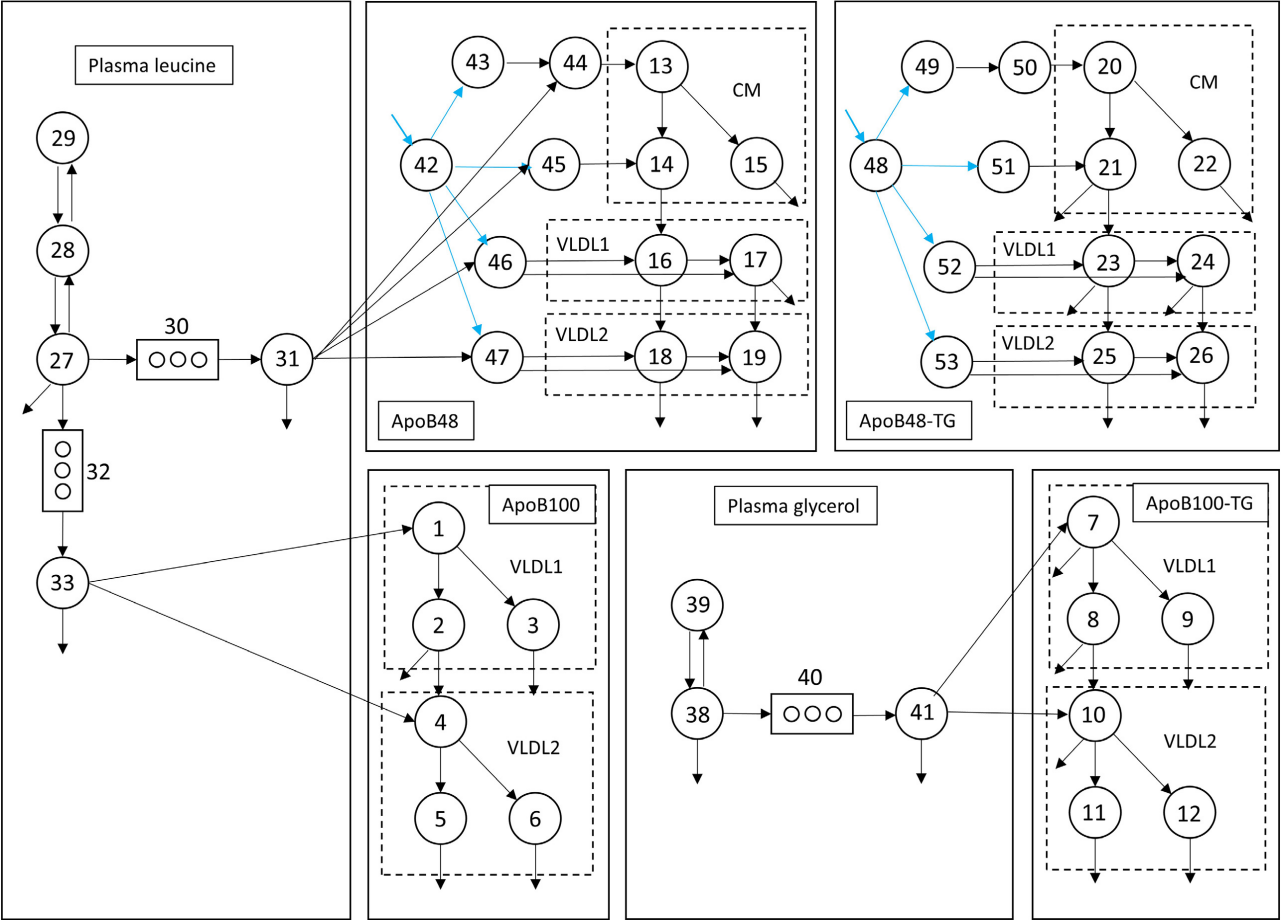
The final integrated model structure. Compartments 1‐6 represents apoB100 in VLDL_1_ and VLDL_2_ fraction; compartments 7–12 represents apoB100‐TG in VLDL_1_ and VLDL_2_ fraction; compartments 13–19 represents apoB48 in CM, VLDL_1_ and VLDL_2_ fraction; compartments 20–26 represents apoB48‐TG in CM, VLDL_1_ and VLDL_2_ fraction. Plasma leucine and plasma glycerol are represented by compartments 27–29 and 38–39, respectively. Blue arrows indicate where postprandial fluxes of apoB48/apoB48‐TG enter the model. The above structure represents a simplified schematic version of the full model (see Figure S2).

The final multicompartmental model, which accounted adequately for the d3‐leucine and d5‐glycerol tracer enrichments and the change in concentration of apoB48 and triglyceride in the CM fraction, and apoB48, apoB100 and triglyceride in the VLDL_1_ and VLDL_2_ fractions, is shown in Fig. 3. As is clear from the data in Fig. 1, it is a non‐steady‐state model in which concentrations change with time in not only the CM fractions but also in VLDL_1_ and VLDL_2_. A complex compartmental structure was required to fit the observations as described for each section of the model below. It should be noted that in a non‐steady‐state analysis, the tracer/tracee ratio (Fig. 1) used as a measure of enrichment in the fitting procedure is a function of both change in tracer (deuterated leucine or glycerol) and tracee (triglyceride, apoB48 or apoB100 mass). Hence, the enrichment and mass data must both be used simultaneously to generate fitted curves.

#### Modelling of plasma leucine and glycerol tracers

Plasma leucine (compartment 27–29, Fig. 3) was modelled as a three compartment subsystem as described previously 5. Compartment 27 represents the compartment where tracer was injected. Compartments 28 and 29 represent tissue pools and interstitial fluid, respectively, in rapid equilibrium with plasma. Plasma d3‐leucine is taken up by the intestine (comp 30) and liver (comp 32) and after a delay (comps 30 + 31; comps 32 + 33), while the apoB peptide chain is synthesized appears in secreted apoB48 (comps 13, 14, 16–19) and apoB100 (comps 1 and 4). Plasma glycerol was modelled as a two compartment subsystem. Compartment 38 represents plasma d5‐glycerol, and compartment 39 represents other rapidly exchanging bodily pools of glycerol. The d5‐glycerol moves via delay compartments 40/41 into the liver where it is incorporated into newly synthesized VLDL_1_‐ and VLDL_2_‐TG. It is assumed that d5‐glycerol does not become incorporated into the triglyceride of intestinally derived lipoproteins since enterocytes possess minimal glycerol kinase activity 25, 26.

#### Modelling of apoB100 and TG in VLDL_1_ and VLDL_2_

Kinetics of apoB100 were described using three compartments in the VLDL_1_ fraction (comps 1, 2, 3) and three in the VLDL_2_ fraction (comps 4, 5, 6) as previously published 5. This allowed for a ‘delipidation chain’ within each lipoprotein fraction; compartment 1 (see Fig. 3) transferred material to compartments 2 and 3 as lipolysis progressed (i.e. as triglyceride was removed from the particle). Compartments 2 and 3 permitted both rapid (comp 2) and slow removal (comp 3) from the VLDL_1_ fraction. A similar structure was employed in VLDL_2_ with compartments 4, 5 and 6.

Triglyceride concentrations and enrichments in VLDL_1_ and VLDL_2_ were modelled as before using a compartmental structure that mirrored that of apoB100 (comps 7, 8 and 9 and comps 10, 11 and 12) 5. Rate constants describing the fluxes to and from compartments 1–6 and 7–12 were allowed to vary freely, but the VLDL_1_/VLDL_2_ apoB100 (comps 1‐6) and VLDL_1_/VLDL_2_‐TG subsystems (comps 7–12 in Fig. 1) were linked by setting the rate constants equal to each other [e.g. *k*(0,5) = *k*(0,11)]. The production rates of apoB100 and VLDL‐TG were also linked to each other. In VLDL_1_, the TG/apoB100 ratio in compartments 1 and 7 were set equal as were the TG/apoB100 ratio in compartments 2, 3, 8 and 9. Likewise in VLDL_2_, the TG/apoB100 ratio was set equal in compartments 4 and 10, and again, the ratio was equal in compartments 5, 6, 11 and 12. The resulting four TG/apoB100 ratios were variables in the model which adjusted according to the rate of delipidation (see Table S1).

#### Modelling of apoB48 in CM, VLDL_1_ and VLDL_2_

Concentrations of apoB48 and triglyceride in apoB48‐containing particles and their tracer enrichments were modelled using three compartments in the CM fraction (13–15 and 20–22) and two compartments in VLDL_1_ (16,17 and 23,34) and VLDL_2_ (18,19 and 25,26). The number of subcompartments was chosen as a compromise between being able to fit the experimental data for a broad range of subjects (normo‐ to hypertriglyceridemic) and having a parsimonious model structure. The CM apoB48 subsystem had three compartments to allow (as in VLDL_1/2_) for a delipidation chain since a newly secreted chylomicron particle likely contains more TG per apoB48 than the average circulating particle in the CM fraction. Accordingly, compartment 13 was allowed to have a higher TG/apoB48 ratio while compartments 14 and 15 were set to have equal, lower TG/apoB48 ratios. Thus, in this model, it is envisaged that newly secreted chylomicrons enter compartment 13 and are delipidated rapidly within the CM fraction to two species with faster (comp 14) and slower (comp 15) ongoing delipidation rates. Further lipolysis leads to the formation of apoB48‐containing remnants first within the VLDL_1_ fraction (comps 16, 17) and then in the VLDL2 fraction (comps 18, 19). The structure of the apoB48 subsystem in the VLDL_1_ and VLDL_2_ fractions (Fig. 1) was a simplified version of the apoB100 model with two compartments in each density interval (comps 16 and 17 represent apoB48 in the VLDL_1_ fraction; comps 18 and 19 represent apoB48 in VLDL_2_) being sufficient to describe the experimental data; there was no requirement for a delipidation chain structure. Compartments 16 and 18 have relatively rapid fractional transfer/clearance rates; compartments 17 and 19 are slower. Inclusion of the latter compartments allow slow decaying species as seen in the tracer curves especially in subjects with higher triglyceride levels. While the TG/apoB48 ratio in the CM fraction is experimentally verifiable, and hence, an adjustable variable in the fitting process the triglyceride associated with apoB48 in the VLDL_1_ and VLDL_2_ ranges is not measured but is connected to the apoB48 model via coupling of rate constants and production rates (as for the apoB100 model). Its kinetics can be simulated as in compartments 20 to 26 and will be a component of the measured triglyceride in VLDL_1_ and VLDL_2_.

#### Postprandial triglyceride secretion

It is established experimentally that intestinal triglyceride absorption is near complete and does not vary substantially between subjects 27, 28, 29. The consequence of this observation for modelling purposes is that total TG secretion in apoB48‐containing particles should largely be determined by the amount of fat in the test meal. Therefore, we assumed a 95% uptake of the dietary fat in the test meal in all subjects. The timing of the apoB48‐TG secretion was, however, free to vary between individuals, and the total apoB48 secretion was not subject to any direct constraints.

### Features of apoB48 and chylomicron metabolism in the integrated model

#### Postprandial apoB48 secretion

In the model when dietary triglyceride is absorbed, it appears first in compartment 48 which represents lipid in the intestinal tract. To account for the subsequent appearance of apoB48 in the bloodstream, two possible modelling structures were tested, one in which all apoB48‐associated lipoprotein secretion in response to the test meal occurred in the CM fraction and could only appear in the VLDL_1_ and VLDL_2_ density ranges through lipolysis, and a second in which direct postprandial secretion of apoB48 lipoproteins into the VLDL_1_ and VLDL_2_ fraction was allowed. The first option did not fit the experimental data well – observed data points fell consistently above the simulated rise curve in both VLDL fractions. When direct secretion was permitted (i.e. transfer from comp 48 to comps 52 and 53 as well as 49 and 51), there was a good fit and so this arrangement was incorporated into the final model (Figs. 3, S2). With this compartmental structure, it was found that in response to the test meal while >95% of absorbed TG appeared in the CM fraction (Fig. 1c,l), a substantial proportion of total apoB48 secretion occurred in the VLDL_1_ and VLDL_2_ fractions (Fig. 1d,e).

#### Basal apoB48 secretion

To assess the level of basal apoB48 secretion (i.e. apoB48 secretion which persists even during prolonged fasting) and to distinguish this from diet‐induced apoB48 secretion, we tested the subjects twice using identical tracer protocols but on one occasion no meal was given. The results of the fasting experiment in terms of total plasma apoB48 concentration and tracer enrichment are presented in Fig. 2. There were measurable concentrations of plasma apoB48 in all four subjects during the prolonged fast, and these levels decreased with time in subjects 3 and 4 where apoB48 was initially high. Subject 1 had low levels throughout. There was clear synthesis and secretion of apoB48 in the fasting state as evidenced by the d3‐leucine enrichment curves. For the four subjects, the mean basal secretion rate during the fasting experiment was 74 ± 29 mg day^−1^ (range 41–108 mg day^−1^) which was not significantly different (*P*‐value = 0.42, two‐tailed *t*‐test) from the estimated rate during the postprandial experiment (53 ± 30 mg day^−1^, range 27–101 mg day^−1^).

#### Modelling the previous day

**Figure 4:**
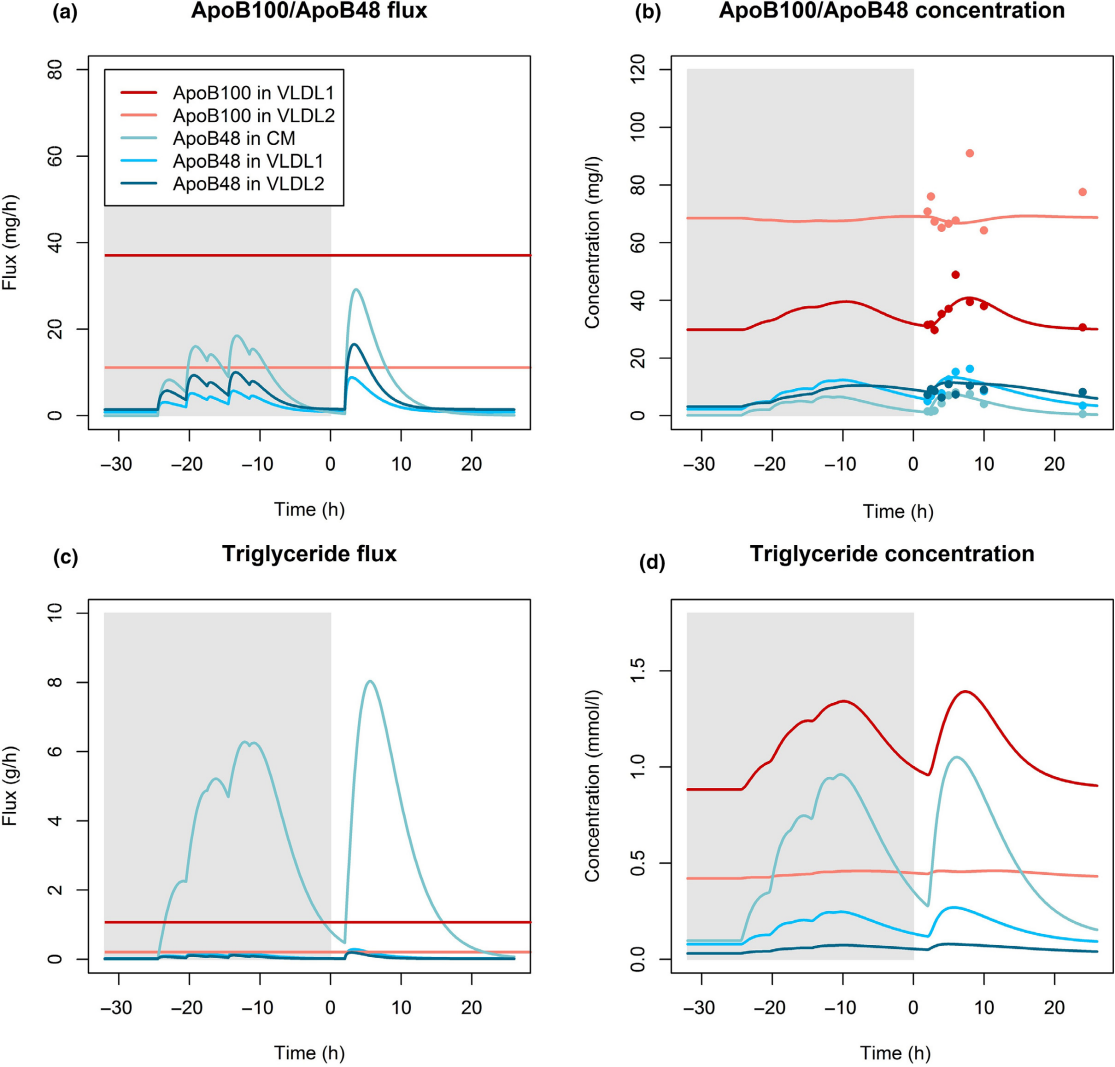
(a) Fluxes of apoB48 in CM, VLDL_1_ and VLDL_2_ and fluxes of apoB100 in VLDL_1_ and VLDL_2_; (b) Concentrations of apoB48 in CM, VLDL_1_ and VLDL_2_ and concentration of apoB100 in VLDL_1_ and VLDL_2_; (c) ApoB48‐TG flux in CM, VLDL_1_ and VLDL_2_ and apoB100‐TG flux in VLDL_1_ and VLDL_2_; (d) ApoB48‐TG concentration in CM, VLDL_1_ and VLDL_2_. Solid lines indicate model predictions and coloured circles indicate experimental data. Modelling of the previous day is indicated with grey background. The concentration and flux (in terms of mass) of apoB100 is higher than that of apoB48 in the VLDL_1/2_ fractions. Total apoB48 flux into the CM fraction is higher than the basal apoB48 flux, and postprandial apoB48 flux also constitutes a significant portion of the total postprandial apoB48 flux. The total triglyceride flux into the CM fraction is the biggest source of triglyceride flux. However, VLDL_1_‐TG concentration is higher than CM‐TG concentration because of the high CM‐TG FCR.

It is clear from the data depicted in Figs 1 and 2 that measurable levels of plasma and VLDL_1_ and VLDL_2_ apoB48 were present even in subjects who had fasted overnight. These particles could result from continuous significant levels of basal secretion and/or the presence of slowly metabolized apoB48‐containing remnants of chylomicron metabolism that persisted for many hours after absorption was complete. The findings of the ‘fasting’ experiment indicated that both of these phenomena occur and it was necessary to accommodate this in the model. Accordingly, a ‘previous day’ was introduced (Figs 2 and 4, shaded area in the panels) to allow for the existence and persistence of these apoB48 particles. In the compartmental model, the particles were assigned the same kinetic behaviour (rate constants) as those measured directly in response to the test meal. Since the exact dietary fat intake was not known, a typical intake was assumed with three main meals (breakfast, lunch, dinner) and two snacks (afternoon and evening). These were modelled with the assumption of a total fat intake of 100 g for the day (for details, see Appendix S1).

### Fluxes and concentrations of apoB48 vs apoB100

In this limited group of subjects, mean basal apoB48 production during the postprandial test day was 53 mg day^−1^ which was small compared to the mean apoB100 production of 1157 mg day^−1^ (range 739–1320 mg day^−1^; Table 2). Mean apoB48 production rate in response to the test meal was 230 mg day^−1^ (range 85–388 mg day^−1^). For a typical day assuming a dietary fat intake of 100 g, this would correspond to around 300 mg of apoB48 secreted per day. This production rate is predicted by the model to vary substantially between subjects, and it will be a function of dietary fat intake. Allowing for the difference in molecular weight between the two apoB proteins, apoB48 particles accounted for about 30% of the total number of secreted TRL particles. It was noteworthy that the FCR of apoB48 in VLDL_1_ and VLDL_2_ density ranges was less than that in the CM fraction and also less than the FCR for apoB100 (Table 2). These observations lend weight to the concept that apoB48 in the VLDL fractions is present on slowly metabolized remnants.

**Table 2:** Individual and mean (±SD) kinetic parameters for the four subjects. Between‐subject variation is mostly evident in several apoB48‐ and apoB100‐related FCR values. Between‐subject variability exists in basal and total apoB48 production rates. All subjects have low basal apoB48‐TG rates. Mean apoB100 production rate in VLDL_1_ and VLDL_2_ is in line with previously published results 5. FCR, fractional catabolic rate, FDC, fractional direct catabolism, FTR, fractional transfer rate, prod, production, dir, direct, tot, total. Postprandial prod refers to the incremental secretion in response to the test meal in addition to the basal secretion. For explanation of calculations of FCR, FDC and FTR, see Appendix S1

	Subject 1	Subject 2	Subject 3	Subject 4	Mean ± SD	Mean ± SD second cohort
ApoB48 kinetic parameters						
ApoB48 tot prod (mg day^−1^)	248	416	142	327	283 ± 100	332 ± 99
ApoB48 basal prod (mg day^−1^)	27.8	27.3	57.4	101	53.4 ± 30	61.8 ± 4.3
ApoB48 postprandial prod (mg day^−1^)	220	388	85	226	230 ± 110	270 ± 96
ApoB48 tot FCR (pools day^−1^)	17.5	27.5	3.0	2.0	12.5 ± 11	6.3 ± 2.2
ApoB48 CM FCR (pools day^−1^)	49.9	26.5	4.5	5.1	21.3 ± 19	24.6 ± 21
ApoB48 VLDL1 FCR (pools day^−1^)	1.5	5.0	1.7	1.5	2.4 ± 1.5	12.3 ± 8.8
ApoB48 VLDL2 FCR (pools day^−1^)	3.8	19	3.0	0.9	6.7 ± 7.2	10.7 ± 2.4
ApoB48‐TG kinetic parameters						
TG apoB48 tot prod (g day^−1^)	67.8	67.1	68	67.7	67.6 ± 0.3	54.8 ± 0.38
TG apoB48 basal prod (g day^−1^)	1.3	0.6	1.5	1.2	1.1 ± 0.3	0.78 ± 0.37
TG apoB48 postprandial prod (g day^−1^)	66.5	66.5	66.5	66.5	66.5 ± 0	53.2 ± 0.37
TG apoB48 CM FCR (pools day^−1^)	238	44	55	23.5	83.4 ± 90	44 ± 15
TG apoB48 VLDL1 FCR (pools day^−1^)	3.4	23.2	2.5	3.1	8.1 ± 8.8	4.6 ± 3.6
TG apoB48 VLDL2 FCR (pools day^−1^)	12.9	23.1	4	1.3	10.3 ± 8.5	5.1 ± 4.0
ApoB100 kinetic parameters						
ApoB100 VLDL1 FCR (pools day^−1^)	51.9	18.7	10.4	1.9	20.7 ± 19	15.3 ± 9.5
ApoB100 VLDL1 FDC (pools day^−1^)	33.1	13.4	9.2	0.6	14.1 ± 12	4.2 ± 3.5
ApoB100 VLDL1 FTR (pools day^−1^)	18.8	5.3	1.1	1.4	6.6 ± 7.2	11.2 ± 7
ApoB100 VLDL1 prod (mg day^−1^)	1080	960	987	559	890 ± 200	710 ± 130
ApoB100 VLDL2 FCR (pools day^−1^)	6.3	3.1	2.8	1	3.3 ± 1.9	3.8 ± 1.4
ApoB100 VLDL2 prod (mg day^−1^)	607	633	418	578	559 ± 84	765 ± 150
ApoB100 VLDL2 dir prod (mg day^−1^)	216	360	312	180	267 ± 72	266 ± 120
ApoB100 VLDL tot prod (mg day^−1^)	1296	1320	1272	739.2	1157 ± 240	976 ± 73
ApoB100‐TG kinetic parameters						
TG apoB100 VLDL1 FCR (pools day^−1^)	58.6	20.4	12.1	2.3	23.4 ± 21	28.3 ± 20
TG apoB100 VLDL1 FDC (pools day^−1^)	43.1	15.3	11.6	1.5	17.9 ± 15	19.4 ± 15
TG apoB100 VLDL1 FTR (pools day^−1^)	15.5	5.1	0.6	0.8	5.5 ± 6.1	9.0 ± 5.7
TG apoB100 VLDL1 prod (g day^−1^)	31.3	27.8	29.1	14.7	25.7 ± 6.5	24.3 ± 11
TG apoB100 VLDL2 FCR (pools day^−1^)	33.8	17.6	4.6	2.8	14.7 ± 12	15.5 ± 10
TG apoB100 VLDL2 prod (g day^−1^)	12.9	16.2	5.3	7.5	10.5 ± 4.3	11.9 ± 2.3
TG apoB100 VLDL2 dir prod (g day^−1^)	4.6	9.2	4	2.3	5.0 ± 2.6	4.2 ± 1.9
TG apoB100 VLDL tot prod (g day^−1^)	35.9	37	33	17.1	31 ± 8	28.4 ± 11

### Dynamics of TG transport in apoB48‐ and apoB100‐containing lipoproteins

The triglyceride content of apoB48‐containing particles in the VLDL fraction was calculated from the apoB48 protein flux and the estimated TG/apoB48 ratio in particles in the VLDL density range. It was predicted to be small compared to the triglyceride content of apoB100‐containing VLDL counterparts, but not negligible. During peak postprandial conditions, roughly 10–20% of the TG in the VLDL‐fraction was predicted to be in apoB48 particles. However, during fasting, this dropped to around 5% or less. The largest discrepancy between flux and concentration is seen for TG in the CM fraction (Fig. 4). Although the TG flux in the CM fraction is around an order of magnitude higher than, for example, the TG in the VLDL_1_ fraction, the concentration ratio is around 1:1 at peak postprandial conditions. Basal TG secretion in apoB48‐containing particles was predicted to be low at about 1.1 g day^−1^ on average (range 0.6–1.5 g day^−1^).

### Validation of model

To validate the model, we performed kinetic studies in four additional overweight or obese subjects. These subjects underwent the same kinetic study protocol. This second cohort had an average plasma triglycerides of 1.3 mmol L^−1^ (range 0.9–1.7 mmol L^−1^) and was selected to be more representative of a normal population in terms of plasma triglycerides than the first cohort which had a wider range of plasma triglycerides. Baseline characteristics of the four additional subjects are shown in Table S2. The kinetic parameters for the first cohort and the second cohort are compared in Figure S5. The results indicate general agreement between the first and second cohort results and indicate future applicability of the model to a ‘normal population’ setting.

## Discussion

Renewed interest in the atherogenic potential of triglyceride‐rich lipoproteins requires deeper insight into how these routes of mass lipid transport are controlled and where dysregulation can lead to increased risk of CHD. Further, recent identification of possible novel therapeutic targets such as apoC3 and ANGPTL3 mandates further exploration of how these and other agencies impact on the complex, integrated pathways of VLDL and chylomicron metabolism 30, 31, 32. The present study offers a new approach to determining the kinetics of triglyceride transport in a setting that more closely reflects normal physiology where across the day there are waves of fat absorption. Arguably until we have good models of chylomicron and apoB48 metabolism, we have limited ability to determine in detail the influence of diet (especially dietary fat) and drugs on this lipoprotein pathway in man.

Key findings from the present study are (i) apoB48 is secreted continuously from the intestine in a basal turnover state at a rate that is less than that reported in previous studies 8, 9, 12, 13, 14, 15, 16, 17, (ii) apoB48 is released in lipoprotein particles across a wide density and size range – from chylomicrons to VLDL_1_ and VLDL_2_, (iii) apoB48 lipoproteins in the VLDL density range can be long‐lived and persist even after a prolonged (overnight) fast, and (iv) a significant proportion of VLDL mass comprises apoB48 particles which have metabolic characteristics that appear to differ from those of apoB100‐containing VLDL. The contribution of this range of apoB48‐containing particles, especially remnants, to the formation of atherosclerotic lesions is yet to be fully elucidated. What is clear is that examination of VLDL kinetics in the fasted state as undertaken by ourselves and others is a convenient approximation to reality that has yielded a great deal of useful information 5, 33, 34, 35, 36. However, the true physiological picture is that of a dynamic system in which VLDL and chylomicron transport are interlinked and both undergo substantial variation in concentration and kinetic behaviour across the day.

Developing an understanding of the metabolism of apoB48 has been challenging due to the low abundance of this protein in the circulation and the requirement to separate it effectively from the large amount of apoB100 present in TRLs. Use of techniques such as gel electrophoresis to isolate the two apoB proteins usually limits investigation to the whole TRL fraction since there is, especially in normal subjects, insufficient material to examine CM and VLDL particles separately. There is also the need to introduce steady‐state‐like conditions if experimental protocols such as primed constant infusion of tracers are used (in distinction to bolus injections). The micro‐meal feeding pattern employed by a number of investigators 8, 10, 11, 18 leads to a relatively constant level of TRL apoB48 across the day and rates of overall apoB48 secretion are reported to be about 73 mg day^−1^ in normal 37 to 644 mg day^−1^ in diabetics 10 with apoB48 FCRs of 4.3 and 3.7 pools day^−1^, respectively. However, this approach cannot distinguish between basal secretion of apoB48‐containing particles during the postabsorptive state and the increment which accompanies fat absorption and this limits interpretation of the findings, although effects of obesity 12 and antidiabetic drugs 10 have been documented. In the initial four subjects examined in the present study, apoB48 total FCR ranged from 27.5 to 2 pools day^−1^, dropping off markedly as plasma triglyceride rose from low normal to high. Total apoB48 production integrated over 24 h was 142 to 416 mg day^−1^.

Seeking to understand better the impact of a fat load on apoB48 metabolism, Wong *et al*. 12, 13 employed (as we did) a test meal and followed the increase in apoB48 mass and tracer enrichment across the day: again, the whole TRL fraction was analysed. They reported incremental rates of apoB48 secretion of about 40–100 mg over 10 h in response to the fat meal and an invariant FCR for TRL apoB48 of about 11–16 pools day^−1^. These values can be compared to the 230 mg day^−1^ (range 85–388 mg day^−1^) increment in our study and a similar average total FCR of 18 pools day^−1^. To account for the observed fasting concentration of apoB48, they invoked basal secretion rates of the order of 340 mg day^−1^ in normal subjects which is very high and implies that the intestine releases several times more apoB48 particles when fat is absent than when it is present; further, if the particles released were of the size of VLDL or chylomicrons, this would imply a basal triglyceride secretion of hundreds of grams which is clearly not a physiologically relevant proposition.

It has been a longstanding observation that significant levels of apoB48 can be detected even after an overnight fast with ‘fasting’ apoB48 reported to be in the order of 5 mg L^−1^ 38, which would correspond to around 5% of all apoB in the VLDL fraction, but with a range from <1 mg L^−1^ up to 60 mg L^−1^ 39, 40, 41, 42, 43, 44, 45. The source and nature of this apoB has been the subject of speculation; is it, as reported in the study of Wong *et al*. 12 the result of basal secretion into the CM size range or into smaller lipoprotein fractions, or is the protein present on chylomicron remnant lipoproteins that have a long residence time? A key observation, which we believe helps resolve this, was the finding in the present investigation that during prolonged fasting where baseline (time 0 h) apoB48 levels were high, the concentration decayed with time and did not approach zero for a further approximately 12 h. Thus, we had to build into the final model the existence of apoB48 particles that had been generated during the previous 24 h (the simulated ‘previous day’). Inclusion of this feature accounted for a significant quantity of ‘fasting’ apoB48, and the basal rates that we observed in this admittedly limited number of subjects were low and hence physiologically plausible.

Recent studies have documented the presence of cytoplasmic lipid droplets in enterocytes before, during and after dietary fat absorption and so care must be taken in interpreting the impetus to continued apoB48 secretion in the ‘fasting’ state 46. These droplets appear to act as a ‘buffer’ to allow rapid fat uptake from the diet and controlled release of lipoprotein particles. Thus, the apoB48 synthesis and secretion we observed in subjects 12 h after food intake could have been associated with lipid‐poor particles 47 or triglyceride transporting particles assembled using lipid stored in cytoplasmic droplets 48. Further metabolic investigations will be required to distinguish between these possibilities.

As mentioned above, many investigators have limited study of apoB48 kinetics to the total TRL density range (*d* < 1.006 kg L^−1^) interval due to the need to isolate sufficient quantities of the protein for analysis. Using advanced mass spectrometric analysis, we were able to quantify the mass and tracer enrichments of apoB48 in the VLDL_1_ and VLDL_2_ fractions. This was an important step forward since it became clear that the kinetic behaviour of apoB48 differed in VLDL compared to CM. It was observed that while the largest source of postprandial apoB48 secretion occurred in the CM fraction, most (around 80% in average for the four subjects) of the apoB48 carrying lipoproteins during a typical day existed in the VLDL density range (Fig. 4). In addition, we observed direct secretion of apoB48 particles into both VLDL_1_ and VLDL_2_ and clearance was also directly from these density intervals. It follows that in many subjects, the intestine through the elaboration and secretion of apoB48 particles is a previously unrecognized contributor to VLDL metabolism in man. About 91 mg day^−1^ (range 26–206 mg day^−1^) of apoB48 was secreted directly into VLDL_1_/VLDL_2_, and the FCR from these density intervals was distinct from that seen for CM apoB48. ApoB48‐containing remnants in TRL especially in individuals with elevated plasma triglyceride levels could therefore conceivably arise from the lipolysis of CM or VLDL_1/2_, and this has implications for understanding the nature of these prime suspects in atherogenesis 1, 2.

The direct entry of apoB48‐containing lipoproteins into the VLDL density range was a key conceptual finding in this study that is supported by previously published data. It is known that chylomicrons can differ in size and density, and it has been proposed that the wide variation in sizes of chylomicrons (35–>250 nm) reflects the supply of triglycerides to the intestine and affords a mechanism by which TG secretion can be readily altered in response to diet [49; NB 35 nm would reach down into the VLDL_2_ size range]. An electron microscopic study conducted by Mahley *et al*. reported that intestinal Golgi vesicles contain either CMs or VLDL particles 50, 51, and it has indeed been proposed that the enterocytes produce mostly apoB48‐VLDL during fasting 52, but following the ingestion of fat, the enterocytes begin to produce the TG‐rich CM particles 50, 53.

This study would not have been possible without the development of advanced liquid chromatography mass spectrometry (LC/MS) analysis. This methodology has several advantages; the sensitivity and specificity are superior to earlier technologies, and it enables information on several protein kinetics to be obtained at the same 20. This minimizes sample volumes required. A weakness with the technology is that it depends on how well the protein is converted into peptides. Incomplete digestion of the protein of interest could contribute to the discrepancies 20. Also, the methodology is technically challenging and expensive and therefore not available to all laboratories.

In conclusion, the advanced mass spectrometry methods and the multicompartment modelling presented here have enabled novel insights into the metabolism of apoB48 in man. Simultaneous investigation with non‐steady‐state modelling of apoB100‐containing and apoB48‐containing lipoproteins in three different density fractions (CM, VLDL_1_ and VLDL_2_), as would occur normally in the postabsorptive state, has been possible. Our findings reveal novel insights into the metabolism of apoB48‐containing particles in VLDL and the concept that the intestine releases apoB48 at a low level in fasting conditions but when dietary fat is present elaborates lipoproteins across a wide size range. Variation in diet fat quantity and quality is likely to influence the nature of the particles generated and with the tools described here, this, and the consequences for the accumulation of potentially atherogenic lipoproteins can be examined with more precision than before.

## Conflict of interest statement

The authors report no duality of interest.

## Contribution statement

The authors contributed to the present work as follows: MRT and JB contributed to conception and design, MA, LA, NM, SS, JK, CS, AT and HZ to the acquisition of data or analysis, and EB, CP, MA, MRT and JB to the interpretation of data. EB and CP drafted the original and revised manuscripts, and all authors approved the final approval of the version to be published.

## Supporting information


**Appendix S1.** Material.
