# Supplementary material for: Investigation of human apoB48 metabolism using a new, integrated non‐steady‐state model of apoB48 and apoB100 kinetics
Source: J Intern Med. 2019 Mar 12;285(5):562–77. doi: 10.1111/joim.12877 (PMC6849847; doi:10.1111/joim.12877)
Supplement: Supplementary file 1 — Appendix S1. Material. [file JOIM-285-562-s001.pdf]

Investigation of human apoB48 metabolism in chylomicron and VLDL subfractions using a new, integrated non-steady-state model of apoB48- and apoB100 kinetics* by Björnson *et al.

SUPPLEMENTARY MATERIAL

Metabolic study protocol. Study subjects participated in two kinetic studies. On the evening of the first visit *(fasting state*), a blood sample was taken. (Supplementary Figure 4). At about 8.00 AM (study 0 h time point), deuterated leucine (5,5,5-D3 Euriso-Top, d3-leucine) at a dose of 7 mg/kg body weight, and deuterated glycerol (1,1,2,3,3-D5 Euriso-Top, d5-glycerol) at an invariant dose of 500 mg (Isotec, Miamisburg, OH) were injected as a bolus. The blood sampling schedule was immediately before tracer injection and then at 2, 4, 6, 8, 10, 12, 15, 20, 30, 45 min and 1, 2, 3, 4, 6, 8, 10 and 24 h for the measurement of d3-leucine tracer concentrations in plasma. Samples taken before the injection of tracers and at 30, 45, 60, 75, 90, 120, and 150 min and 3, 4, 5, 6, 8, 10 and 24 h after were used for the measurement of d3-leucine enrichment in apoB48 and apoB100 and d5-glycerol enrichment in triglyceride in VLDL1 and VLDL2 fractions.

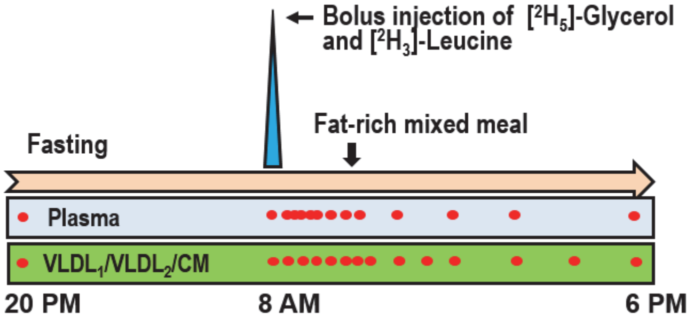

***Supplementary figure 1.** Protocol of kinetic study. Red dots indicate blood sampling.*

ApoB48 quantification using mass spectrometry. Since apoB48 is a low-abundant protein, we utilized antipeptide antibodies to enrich apoB48 and from digested samples, namely the stable-isotope-labeled standards and capture by antipeptide antibody approach.[1] The C-terminal tryptic peptide LSQLQTYMI for ApoB48 is the only peptide that distinguish ApoB48 from ApoB100. The main advantage of quantification using parallel reaction monitoring (PRM) on a high resolution/accurate mass (HR/AM) instrument over low resolution multiple reaction monitoring/selected reaction monitoring (MRM/SRM) quantification is the high selectivity in the analysis. Furthermore, all fragment ions are detected in parallel and no prior selection has to be performed. The fragment ions used in the ApoB48 kinetic studies contain one d3-leucine. The high resolution and high accuracy of the analysis allow the differentiation between d3-leucine fragment ions from the fragments of the d0 M3 isotope at low levels of incorporation.[2] The LC conditions for separation and MS parameters were optimization to enhance the detection of the fragment ions containing d3-leucine. For absolute quantification of ApoB48 and its oxidized form the corresponding stable-isotope-labeled peptides were used as internal standards (ISTDs).

ApoB48 was quantified in plasma and in the CM, VLDL1 and VLDL2 fractions. The level measured in whole plasma was routinely found to be less than the sum of the absolute measurements in the individual fractions from each subject and time point. On average we found apoB48 in the summed fractions to be 1.4 times the plasma level and this value was applied as a scaling factor in the model to resolve the discrepancy during the fitting process.

[1] Anderson NL, Anderson NG, Haines LR, Hardie DB, Olafson RW, Pearson TW. Mass spectrometric quantitation of peptides and proteins using Stable Isotope Standards and Capture by Anti-Peptide Antibodies (SISCAPA). *J Proteome Res* 2004; 3: 235-44.

[2] Singh SA, Andraski AB, Pieper B, Goh W, Mendivil CO, Sacks FM, Aikawa M. Multiple apolipoprotein kinetics measured in human HDL by high-resolution/accurate mass parallel reaction monitoring. *J Lipid Res* 2016; **57:** 714-28.

Parallel reaction monitoring for absolute quantitation. The PRM analyses were performed using a Q-Exactive mass spectrometer interfaced to an Easy-nLC 1200 (Thermo Fisher Scientific). The LC conditions for separation and MS parameters were optimised to enhance the detection of the fragment ions containing d3-leucine for both the ApoB48-peptide and its oxidized form. Peptides were trapped on an Acclaim Pepmap 100 C18 trap column (100 µm x 2 cm, particle size 5 µm, Thermo Fischer Scientific) and separated on an in-house constructed analytical column (150x0.075 mm I.D.) packed with 3 µm Reprosil-Pur C18-AQ particles (Dr. Maisch, Germany) using a linear gradient from 17% to 50% B over 17 min followed by an increase to 100% B for 3 min and a washing step at a flow of 300 nL/min. Solvent A was 0.2% formic acid in water and solvent B was 80% acetonitrile in 0.2% formic acid. Ions were injected into the mass spectrometer under a spray voltage of 1.8 kV in positive ion mode. For the PRM method, an orbitrap resolution of 35 000 (at m/z 200), a target AGC value of 2e6, a maximum fill time of 110 ms, and a quadrupole isolation window of 2 Th were used. The precursor ions of the endogenous, the D3endogenous and labeled peptides were targeted at their 2+ charge without scheduling. Fragmentation was performed with normalized collision energy (NCE) of 10.

Raw mass spectrometry data (Thermo) were exported to Skyline (version 3.7) for extraction of the fragment ions and peak area integration. The integration boundaries were manually verified for all peaks in the samples. A list of the precursor ions for the corresponding pairs of endogenous, d3-endogenous and labeled peptides and selected fragment ions is given in **Supplement Table 4**. All the ion fragments used for determine turnover and the ratio of endogenous and d3-endogenous contained d3-leucine.

To determine the linearity of the ISTD peptides in the standard curve samples a regression analysis was performed on the individual fragments from the two peptides. The MS response was found to be linear within the required concentration range for both peptides. The ISTD peptides were spiked in the individual plasma samples at concentrations close to those determined for their endogenous counterparts in the pool of samples analyzed in the standard curve. The concentration of the endogenous peptides in the individual study samples were calculated by multiplying the concentration of the labeled peptide (4.17, 12.5 and 11.9 fmol/µL in CM-fraction, VLDL1/2-fraction and in plasma respectively) with the Ratio To Standard. Concentrations in mg/l were calculated assuming a molecular weight of apoB48 of 243 kDa.

SAAM modeling. The most commonly used software for kinetic analysis in the field of lipoprotein metabolism is the Simulation Analysis And Modeling (SAAM) program (https://tegvirginia.com/software/saam-ii) which via a graphical interface allows the user to define easily complex compartmental models. Each compartment represents an entity with defined input and decay rates (a rate constant within an ordinary differential equation). Where a lipoprotein fraction exhibits complex (multi-exponential) kinetics, compartments (differential equations) are grouped together to provide simulated outputs that can be fitted to the observed data. Data entered into SAAM includes, for stable isotope experiments, the amount of tracer administered, tracer enrichments in each lipoprotein fraction, and observed lipoprotein masses (in our case apoB48 and apoB100 circulating pool sizes in mg). The program undertakes through an iterative process to minimize the difference between prediction and observation and so achieve an optimised fit. When the best possible fit is obtained within the limits of the model structure and data measurement error then the program outputs a rate constant for each mathematical compartment describing the rate of change with time (e.g. for compartment 1 in Figure 3), aggregate fractional catabolic (clearance) rates for the compartments that together make up the lipoprotein fraction, for example compartments 1, 2 and 3 for VLDL1 apoB100, a mass for the aggregated compartments and by calculation a production rate (mass multiplied by the fractional catabolic rate). Validation of the model is based on the achievement of a global minimum to the sum of squares of the differences between observed and predicated data values (this is when the SAAM program outputs its results), no systematic bias indicating an inadequate model structure (the observed data scatter around the predicted line due to measurement error), and acceptable precision of the rate

constants. The last is a function of the goodness-of-fit of the model and the measurement error within the data. A rate constant can have a high coefficient of variation due to 'noisy' data but be acceptably well defined if the fit is optimized.

Previous day fat intake estimations. Since dietary records were not kept during the days before the test days an estimate of the subject's fat intake was made. The subject's daily energy expenditure was estimated using National Institute of Health Body Weight Planner (https://www.niddk.nih.gov/bwp) and their average anthropometric measurements. A daily physical activity level of 1.6 was assumed. The subjects were further assumed to be in energy balance and consume 1/3 of calories as dietary fat which corresponds to 100 grams of daily fat intake. The subjects were assumed to consume breakfast at 07:30, lunch at 11:30, a snack at 2:30 pm, dinner at 5:30 pm and an evening snack at 8:30 pm the day before the test days. The fat content of the individual meals was assumed to be 20 g, 30 g, 10 g, 30 g and 10 g respectively. These estimations were considered physiologically plausible.

Assumptions of large precursor pool. The precursor pools for apoB48 and apoB100 (i.e. pools of leucine to be incorporated into apoB100 and apoB48) are assumed to be large enough for the contribution of the synthesis of apoB100 and apoB48 to not affect the pool size. For this reason, the apoB100 and apoB48 production rates could be modeled as external fluxes (U(x), U(y) etc).

Definition of FCR, FDC and FTR. The fractional catabolic rates (FCRs) of apoB100 have been calculated by summing the accumulated daily steady-state flux into and out of a fraction (e.g. $VLDL_1$) and dividing by the steady-state pool size (of e.g. apoB100) in that fraction. This produces FCRs in the unit pools/day. For example the apoB100 FCR in $VLDL_1$ have been calculated by:

$$apoB100\,VLDL1\,FCR = (FLUX(0,2) + FLUX(4,2) + FLUX(0,3)) * 24 / (Q1 + Q2 + Q3)$$

where compartment numbers (1-4) refer to Figure 3 in the main text and fluxes are expressed in mg/h and pool sizes (Q) are expressed in mg. The FDC (fractional direct removal) of apoB100 in $VLDL_1$ is defined by:

$$apoB100\,VLDL1\,FDC = (FLUX(0,2) + FLUX(0,3)) * 24 / (Q1 + Q2 + Q3)$$

And thus refer to the number of pools per day which is "directly removed" from the $VLDL_1$ fraction and not transferred to the $VLDL_2$ fraction. Conversely, the apoB100 $VLDL_1$ FTR (fractional transfer rate) is defined by:

$$apoB100\,VLDL1\,FTR = (FLUX(4,2)) * 24 / (Q1 + Q2 + Q3)$$

And thus refer to the number of pools per day which is transferred from $VLDL_1$ to $VLDL_2$ and not "directly removed".

Since apoB48 is not close to being in steady state the same calculations cannot be applied. Instead the apoB48 FCR values was calculated by summing (the variable) fluxes throughout the 24 hour test-day period and dividing by the average pool size throughout the same 24 hour period. Thus the apoB48 FCR values are sensitive to dietary fat intake and should be interpreted in this context.

Direct production of apoB48-containing lipoproteins into VLDL density range. In our Initial model, in line with the prevailing paradigm, apoB48 production was solely into the CM fraction and any apoB48 appearing in VLDL1 and VLDL2 was the result of lipolysis. With the Initial model, as can be seen in panels A and B the model-predicted line consistently failed to fit (fell below) the observed early rise in enrichment in both VLDL fractions. In the Final model direct input of apoB48 into VLDL1 and VLDL2 in addition to CM was allowed, and this resulted in a good fit to the experimental data (see panel C and D). Indeed, in all subjects examined the enrichment in apoB48 rose virtually simultaneously in CM, VLDL1 and VLDL2 (Panel G). This simultaneous rise pattern was predicted by the Final model version (F) better than the Initial model version (E).

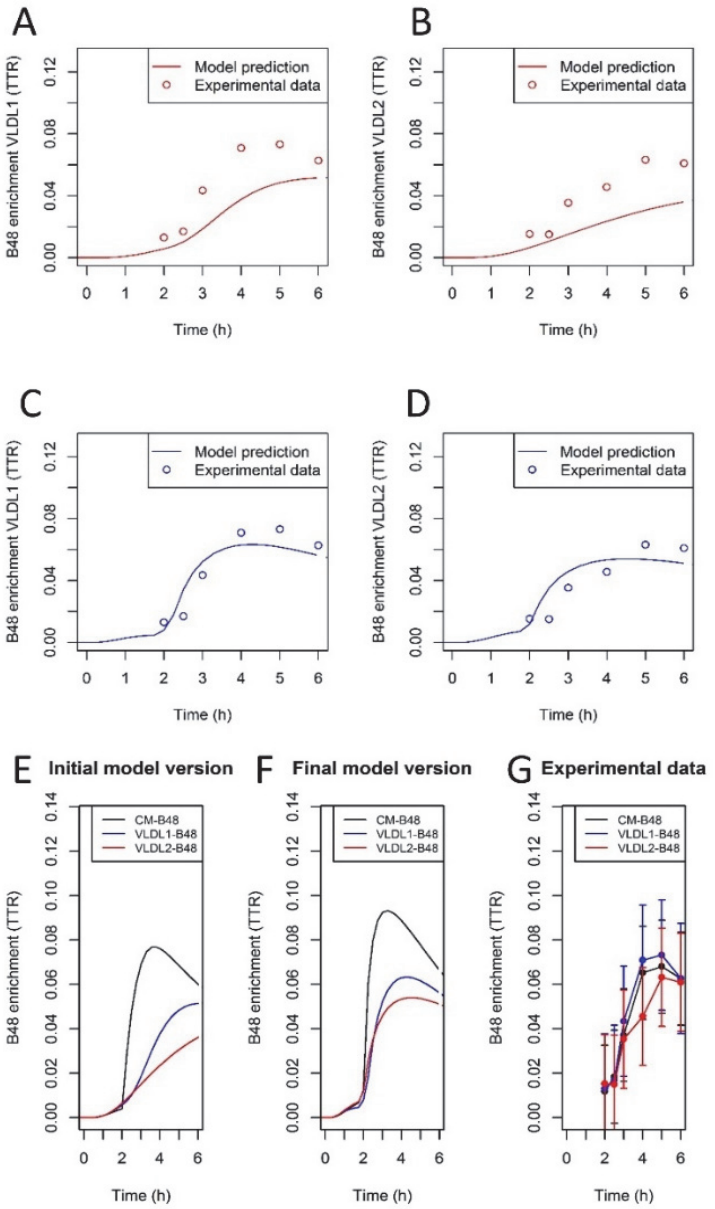

Supplementary Figure 2. *Model generated predicted enrichment curves using the Final model with direct production of apoB48 into VLDL1 and VLDL2 gave a rise in apoB48 enrichment in VLDL1- and VLDL2 that agreed with experimental data (panel C, D). This fit can be compared with the Initial model predictions of apoB48 enrichment in VLDL1- and VLDL2 (panel A, B) which show slower rise in enrichment due to the fact that apoB48 reaches these density intervals only as a result of sequential transfer (i.e. delipidation from CM to VLDL1 and then VLDL2) as shown in panel E. The Final model (panel F) permitted a simultaneous rise in apoB48 enrichment in CM, VLDL1 and VLDL2, a pattern more consistent with the experimental data (panel G which depicts the mean +/- SD observed enrichment in CM, VLDL1 and VLDL2).*

Parameter variance. The final multi-compartmental model was large and complex due to the comprehensive range of data included in the integrated scheme. Visual inspection of the model-predicted curves and observed data is a good guide to the quality of the fit and hence the applicability of the multi-compartmental model structure. Observed and fitted data are provided for all 8 subjects in this Supplement and it can be seen in the main that there is no systematic bias using the Final model, rather there is within subject scatter around the fitted line especially for lipoprotein fractions where technical challenges limited the precision of the acquired data.

Due to the nature of the data, and the mixed steady-state: non-steady state features of the multicompartmental model, we had to use a combination of approaches to gauge the variance of the generated kinetic parameters. Where possible the precision of key parameters – median coefficients of variation of production rates and fractional catabolic rates - was determined using the SAAM software itself (SAAM is able to generate coefficients of variation for steady-state rates automatically). To estimate the variance of non-steady state rates, primarily those associated with apoB48 kinetic during the fat meal test, we calculated coefficients of variation based on the relative precision of the individual

rate constants used in the determination of each production or fractional clearance rate. Most apoB100-related kinetic parameters show relatively low variance whereas apoB48-related kinetic parameters show higher variance. The highest coefficient of variation was found for basal production of apoB48 during the experimental day when the fat meal was given. In this context the results from the fasting-day experiment were helpful since similar basal production rates were estimated without the interference from the meal.

Kinetic parameter	*Coefficient of variation category*
ApoB48 Tot prod	*
ApoB100 VLDL tot prod	*
ApoB100 VLDL1 prod	*
ApoB100 VLDL2 dir prod	*
ApoB100 VLDL2 FCR	*
TG apoB100 VLDL tot Prod	*
TG apoB100 VLDL1 prod	*
TG apoB100 VLDL2 dir prod	*
TG apoB100 VLDL2 FCR	*
ApoB48 basal prod	***
ApoB48 postprandial prod	**
ApoB48 Total FCR	**

Supplementary Table 1: *Coefficient of variation for the key kinetic parameters. The coefficient of variation of each kinetic parameter for the first cohort (n=4) are presented. One star (*) indicate a coefficient of variation of < 50 %, two stars (**) indicate coefficient of variation of between 50 % and 100 % and three stars (***) indicate coefficient of variation of > 100 %. Note CVs are a function of goodness-of-fit of the model and measurement error in the data.*

Basal apoB48 production and comparisons with previous models. In previous models [9-10, 12-14] basal production of apoB48 has been reported to be substantially higher than in the present study; instead of the average 53 mg/day of basal apoB48 production that we found using our Final model, values as high as 300-500 mg/day [12] have been reported.

There are two main differences in the conceptual approach and structure of the multicompartmental model which account for these discordant results; i) previous models only included a single apoB48 compartment and ii) previous models have not taken into account the possible existence of a significant pool of slowly decaying apoB48 particles generated during previous fat intake and persisting in the circulation overnight and into the experimental period of observation (**Suppl. Fig 2**). Including only one apoB48 compartment restricts the model in the sense that it cannot distinguish between rapidly cleared apoB48 in the CM fraction and slowly cleared apoB48 in the VLDL fractions (as we see is the case in the present study). In order to then be able to fit the postprandial excursion curve the fractional clearance rate of the single apoB48 compartment (representing all apoB48 species) needs to be high. This in turn leads to the calculation of a high basal secretion (i.e. measured plasma level of apoB48 in the fasting state before giving the test meal multiplied by the high fractional clearance rate). In the present study we could experimentally distinguish between apoB48 in the CM, VLDL1- and VLDL2 fraction (which previous reports have not been able to do) and so estimate individual fractional clearance rates for each fraction. It was noted that the basal apoB48 was found almost exclusively in VLDL1- and VLDL2 but not in the CM fraction. Thus, high basal production of rapidly turning over apoB48 was not needed to account for fasting apoB48, and the model generated lower basal apoB48 production.

In addition to a more nuanced experimental and modelling approach we also measured apoB48 levels across a day when the subject was examined using a similar tracer protocol but no fat meal was given. We observed especially in subjects with high basal apoB48 (high plasma triglyceride) a decay of apoB48 throughout the day indicating the presence of persistent and slowly removed lipoprotein species. (**Suppl. Fig 3**). Thus, by taking the ‘previous day’ into account we could estimate the basal apoB48 production rate with better precision.

Previous studies using single compartment models that did not take into account persistence of slowly-decaying apoB48 particles estimated basal apoB48 production to be in the order of 300-500 mg/day (9-10, 12-14). This implies that the intestine (allowing for the 2-fold difference in molecular weight in apoB48 compared to apoB100) releases apoB48 particles at a similar rate to liver production of VLDL apoB100 particles even during fasting conditions, which does not seem to be plausible. Our results rather suggest that the intestine during fasting conditions produces in the order of 5-20 % of the number of particles that the liver produces which is more likely to fit with known physiology.

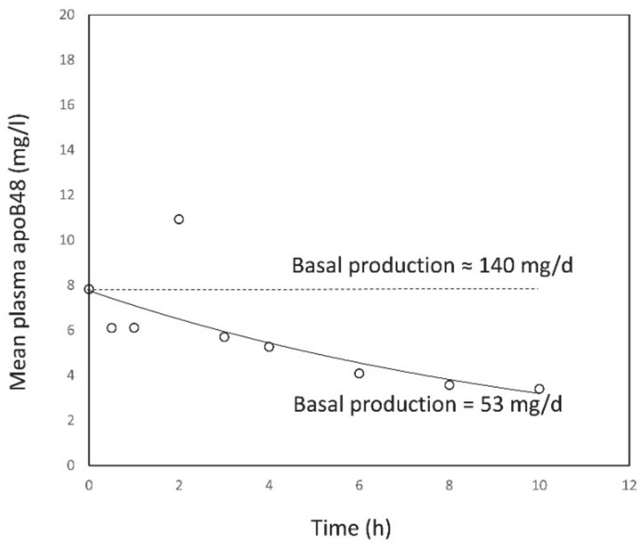

Supplementary Figure 3: *Mean plasma apoB48 levels of the four subjects across the prolonged fasting test day. It was seen that apoB48 decreased over the 10 hours of the observation period which is consistent with the presence of a slowly decaying pool of apoB48 generated from the previous day's meal intake (open circles). The black dotted line indicates the theoretical steady-state concentration resulting from a high level of continuous basal secretion of apoB48 of 140mg/d which is what would be needed to account for the time zero concentration in the absence of any pre-existing apoB48 particles. The solid black line is an exponential regression fit to the experimental data which is best explained by a much lower mean basal production rate of 53 mg/d and the persistence of apoB48 particles from the previous day with a VLDL1/VLDL2 apoB48 FCR of about 2 to 6 pools/day (see Main text,* ***Table 2****)*

Strengths and limitations. The strengths in our experimental approach relate to the use of multiple stable isotope tracers, advanced mass spectrometry techniques and the development of a successful, integrated multi-compartmental model. Limitations relate to the protocol (choice of data time points) and the use of a single experimental standard meal (as opposed to a traditional 3 or 4 meal intake). The approach we took was pragmatic and acceptable to participants. The complexity of the multi-compartmental model that was developed reflected the comprehensive nature of the data and of necessity the model included a large number of rate constants and other parameters such as non-steady state masses (i.e. in plasma, CM, VLDL1 and VLDL2 apoB48, apoB100, triglycerides). The rule of parsimony was employed in that compartments, inputs and outputs were only added to the model if the predicted outputs failed systematically to fit the observed data (see Supplementary figure 1 as an example). Some rate constants were not well defined but the precision of the major global parameters was acceptable (**Supplementary Table 2**). Our approach to estimation of non-steady-state variables was to derive 'aggregate values' over a day. Other models were considered including permitting high basal apoB48 secretion rates to account for apoB48 observed in the fasting condition as used by e.g. Wong et al [12]. As set out in the text, this resulted in non-physiological levels of triglyceride release from the intestine.

Validation in a further set of subjects. In order to validate the model, we recruited an additional four subjects. These subjects underwent the same experimental protocol. The second cohort had an average plasma TG of 1.3 mmol/l (range 0.9-1.7 mmol/l) and was selected to be more representative of a normal population in terms of plasma triglycerides than the first cohort which had a wider range of plasma triglycerides.

	Subject 5	Subject 6	Subject 7	Subject 8	Mean ± SD
Age (y)	60	64	64	54	60.5 ± 4.1
Body weight (kg)	93.8	95.9	97.5	95.8	95.8 ± 1.3
BMI (kg/m²)	27.4	32	28.2	29.2	29.2 ± 1.7
Waist (cm)	103	118	107.5	111	110 ± 5.5
Triglycerides (mmol/L)	0.9	1.27	1.45	1.71	1.33 ± 0.29
ApoC-III (mg/dl)	4.15	13.53	9.7	6.61	8.5 ± 3.5
Total chol (mmol/l)	4.77	5.64	4.58	4.9	4.97 ± 0.4
LDL chol (mmol/L)	3.47	4.47	3.67	3.89	3.88 ± 0.37
HDL chol (mmol/L)	1.33	1.41	0.87	0.77	1.1 ± 0.28
FFA (µmol/l)	671	563	521	571	582 ± 55
LPL activity (mU/ml)	182	172	90.7	156	150 ± 36
HL activity (mU/ml)	206	120	273	231	208 ± 56

Supplementary Table 2: *Baseline characteristics of the four additional subjects included in the validation set. Subjects were overweight or obese but normotriglyceridemic.*

The subjects were fitted to the experimental data and the resulting kinetic parameters are found in **Supplementary Table 5**. The 1st and 2nd cohort's results generally look similar. The model fits are generally acceptable and look similar to the fits for the first cohort.

Supplementary Table 3: List of dependencies. K indicates rate constants, U indicates fluxes of basal apoB48- and apoB100 secretion rates and s indicates how the experimental data and the model predictions are linked. The rate constant refer to the full model nomenclature displayed in **Supplementary Figure 6** and not the schematic model structure displayed in **Figure 1** in the main text.

**LIST OF EQUATIONS AND DEPENDENCIES**
k(1,49) = (B48_rat_CM1L/B48_rat_CM1S)*k(0,49)
k(47,25) = (B100_rat_V2L/B100_rat_V2S)*k_0_25
k(20,30) = k_0_44_slow*((Vmax)/(Vmax + flux(49,53).1))
k(24,44) = (k_0_44_slow*(B100_rat_V1L/B100_rat_V1S))*((Vmax)/(Vmax + flux(49,53).1))
k(23,44) = (k_0_44*(B100_rat_V1L/B100_rat_V1S))*((Vmax)/(Vmax + flux(49,53).1))
k(19,30) = k_0_44*((Vmax)/(Vmax + flux(49,53).1))
k(10,9)=(B48_ratio_CM2/B48_ratio_CM3)*k_0_9
k(26,25) = k_0_25_slow*(B100_rat_V2L/B100_rat_V2S)
k(25,23)=(k_0_23*(B100_rat_V1S/B100_rat_V2L))*((Vmax)/(Vmax + flux(49,53).1))
k(21,19)=k_0_23*((Vmax)/(Vmax + flux(49,53).1))
k(3,2)=(B48_ratio_CM2/B48_ratio_CM3)*k(0,2)
k(2,1)=(B48_rat_CM1S/B48_ratio_CM2)*k(0,1)
k(0,44)=(1 - (B100_rat_V1L/B100_rat_V1S))*(k_0_44 + k_0_44_slow)*((Vmax)/(Vmax + flux(49,53).1))
k(0,19) = k_0_19*((Vmax)/(Vmax + flux(49,53).1))
k(0,9)=k(0,7) + ((1 - (B48_ratio_CM2/B48_ratio_CM3))*k_0_9)
k(0,23)=(k_0_19 + ((1 - (B100_rat_V1S/B100_rat_V2L))*k_0_23))*((Vmax)/(Vmax + flux(49,53).1))
k(0,25)=((1 - (B100_rat_V2L/B100_rat_V2S))*(k_0_25 + k_0_25_slow))
U(43)=U(52)/B48_ratio_CM3
U(42)=U(51)/B48_ratio_CM2
U(52)=B48_CM3_PR1
U(51)=B48_CM2_PR1
U(58)=U(56)/B48_ratio_CM3
U(57)=U(55)/B48_ratio_CM2
U(56)=B48_CM3_PR2
U(55)=B48_CM2_PR2
U(44)=U(30)/B100_rat_V1L
U(30)=B100_V1_PR
U(21)=B100_V2_PR
s23 = (q8 + q6 + q7 + q5 + q13 + q4 + q48)/(q8 + q6 + q7 + q5 + q13 + q4 + q48 + Q8+Q6+Q7+Q5+Q13+Q4+Q48 + q8.1 + q6.1 + q7.1 + q5.1 + q13.1 + q4.1 + q48.1)
s22 = (Q26 + Q47 + Q25 + q26.1 + q47.1 + q25.1)/Blood_vol
s21 = (Q24 + Q23 + Q44 + q24.1 + q23.1 + q44.1)/Blood_vol
s19 = (Q10 + Q3 + q10.1 + q3.1)/Blood_vol
s20 = (Q9 + Q2 + q9.1 + q2.1)/Blood_vol
s14 = (q46+q21 + q22)/(Q46+Q21+Q22+q46+q21+q22)
s12 = (q30+q19 + q20)/(Q30+Q19+Q20+q19+q20+q30)
s18=(Q48 + q48.1 +Q8+q8.1+Q6+q6.1+Q7+q7.1+Q5+q5.1+Q13+q13.1+Q4+q4.1)/Blood_vol
s17=(scalar_CM_TG*(Q1 + q1.1 + Q18 + q18.1+Q49+q49.1))/Blood_vol + (Q47+Q2+q2.1+Q9+q9.1+Q10+q10.1+Q3+q3.1+Q26+q26.1+Q25+q25.1+Q24+q24.1+Q23+q23.1)/Blood_vol+missing_TG
s16 = (q47+q26 + q25)/(Q47+Q26 + Q25+q47+q26+q25)
s15 = (q44+q24 + q23)/(Q44+Q23 + Q24+q44+q23+q24)
s11 = (Q44+Q2 + Q9 + Q24 + Q23 + q2.1 + q9.1 + q24.1 + q23.1)/Blood_vol
s13 = (Q47+Q3 + Q10 + Q26 + Q25 + q3.1 + q10.1 + q26.1 + q25.1)/Blood_vol
s10 = (Q22 + Q21+Q46)/Blood_vol
s9 = (Q30 + Q20 + Q19)/Blood_vol
s5=(q4+q13+q48)/(q4+q13+q48+Q4+q4.1+Q13+q13.1+Q48+q48.1)
s4=scalar_CM_B48*(Q18+Q1+q1.1+q18.1+Q49+q49.1)/Blood_vol
s1=scalar_CM_B48*(Q13+Q4+q4.1+q13.1+Q48+q48.1)/Blood_vol
s8 = (q11/(q11+Q11))*100
s7 = (q8 + q6)/(q8 + q6+Q8+Q6+q8.1+q6.1)

s6 = (q7 + q5)/(q7 + q5+Q7+Q5+q7.1+q5.1)
s3 = scalar_V2_B48*(Q8 + Q6 + q8.1 + q6.1)/Blood_vol
s2 = scalar_V1_B48*(Q7 + Q5 + q7.1 + q5.1)/Blood_vol

Supplementary Table 4: Precursor ions and their corresponding ion fragments used for quantification and determination of turnover by calculation of endogenous and d3-endogenous ratio.

	**B48**	**B48 d3**		**B48MetOx**	**B48MetOx d3**
**Peptide (precursor)**	548.788992, 2+	550.298407, 2+		556.786449, 2+	558.295864, 2+
**b6**	671.372266	674.391096	b5	570.324588	573.343418
**b7**	834.435595	837.454425	b6	671.372266	674.391096
**b8**	965.476079	968.494909	b7	834.435595	837.454425

	**B48**	**B48 iSTD**	**B48MetOx**	**B48MetOx iSTD**
**Peptide (precursor)**	548.788992, 2+	552.297574, 2+	556.786449, 2+	560.295031, 2+
**b5**	570.324588	570.324588	570.324588	570.324588
**b6**	671.372266	671.372266	671.372266	671.372266
**b7**	834.435595	834.435595	834.435595	834.435595
**b8**	965.476079	965.476079	981.470994	981.470994

	1st Cohort					2nd Cohort					
	**Subject 1**	**Subject 2**	**Subject 3**	**Subject 4**	**Mean ± SD**	**Subject 5**	**Subject 6**	**Subject 7**	**Subject 8**	**Mean**	**P-value**
ApoB48 Tot prod (mg/day)	248	416	142	327	283 ± 100	181	322	454	369	332 ± 99	0.58
ApoB48 Basal prod (mg/day)	27.8	27.3	57.4	101	53.4 ± 30	55.2	60.8	64.3	66.7	61.8 ± 4.3	0.66
ApoB48 Postprandial prod (mg/day)	220	388	85	226	230 ± 110	125	261	390	303	270 ± 96	0.65
ApoB48 Tot FCR (pools/day)	17.5	27.5	3	2	12.5 ± 11	8.27	8.55	4.79	3.56	6.29 ± 2.2	0.39
ApoB48 CM FCR (pools/day)	49.9	26.5	4.5	5.1	21.5 ± 19	53.4	35.6	4.15	5.32	24.6 ± 21	0.85
ApoB48 VLDL1 FCR (pools/day)	1.5	5	1.7	1.5	2.43 ± 1.5	4.45	27.1	7.87	9.64	12.3 ± 8.8	0.15
ApoB48 VLDL2 FCR (pools/day)	3.8	19	3	0.9	6.68 ± 7.2	12	12.8	11.2	6.7	10.7 ± 2.4	0.42
ApoB100 VLDL tot prod (mg/day)	1296	1320	1272	739.2	1160 ± 240	1070	951	873	1010	976 ± 73	0.29
ApoB100 VLDL1 prod (mg/day)	1080	960	987	559	897 ± 200	738	891	537	672	710 ± 130	0.23
ApoB100 VLDL1 FCR (pools/day)	51.9	18.7	10.4	1.9	20.7 ± 19	18.8	29.3	6.71	6.51	15.3 ± 9.5	0.68
ApoB100 VLDL1 FDC (pools/day)	33.1	13.4	9.2	0.6	14.1 ± 12	2.3	9.84	0.673	3.89	4.18 ± 3.5	0.25
ApoB100 VLDL1 FTR (pools/day)	18.8	5.3	1.1	1.4	6.65 ± 7.2	16.5	19.5	6.04	2.62	11.2 ± 7	0.47
ApoB100 VLDL2 prod (mg/day)	607	633	418	578	559 ± 84	984	651	819	606	765 ± 150	0.095
ApoB100 VLDL2 dir prod (mg/day)	216	360	312	180	267 ± 72	330	59.9	340	336	266 ± 120	1
ApoB100 VLDL2 FCR (pools/day)	6.3	3.1	2.8	1	3.3 ± 1.9	6.27	3.15	3.09	2.75	3.82 ± 1.4	0.72
TG apoB48 Tot prod (g/day)	67.8	67.1	68	67.7	67.7 ± 0.34	55.4	54.6	54.5	54.5	54.8 ± 0.38	<0.0001
TG apoB48 Basal prod (g/day)	1.3	0.6	1.5	1.2	1.15 ± 0.34	1.42	0.635	0.535	0.544	0.78 ± 0.37	0.25
TG apoB100 VLDL tot Prod (g/day)	35.9	37	33	17.1	30.8 ± 8	41.8	23.5	14.1	17.7	24.3 ± 11	0.43
TG apoB100 VLDL1 prod (g/day)	31.3	27.8	29.1	14.7	25.7 ± 6.5	47	24.4	19.4	22.9	28.4 ± 11	0.73
TG apoB100 VLDL1 FCR (pools/day)	58.6	20.4	12.1	2.3	23.4 ± 21	56	39.7	9.21	8.47	28.3 ± 20	0.78
TG apoB100 VLDL1 FDC (pools/day)	43.1	15.3	11.6	1.5	17.9 ± 15	42.5	24.2	4.33	6.47	19.4 ± 15	0.91
TG apoB100 VLDL1 FTR (pools/day)	15.5	5.1	0.6	0.8	5.5 ± 6	13.5	15.5	4.88	2.01	8.97 ± 5.7	0.5
TG apoB100 VLDL2 prod (g/day)	12.9	16.2	5.3	7.5	10.5 ± 4.3	15.3	10.1	12.7	9.41	11.9 ± 2.3	0.64
TG apoB100 VLDL2 dir prod (g/day)	4.6	9.2	4	2.3	5.03 ± 2.6	5.22	0.93	5.22	5.22	4.15 ± 1.9	0.65
TG apoB100 VLDL2 FCR (pools/day)	33.8	17.6	4.6	2.8	14.7 ± 12	33.3	10.3	9.97	8.45	15.5 ± 10	0.93

Supplementary table 5: *The kinetic parameters for the 1st cohort is compared to the 2nd cohort. The results indicate general agreement between the 1st and 2nd cohort results and indicate future applicability of the model to a "normal population" setting. P-values were calculated using two-tailed t-test.*

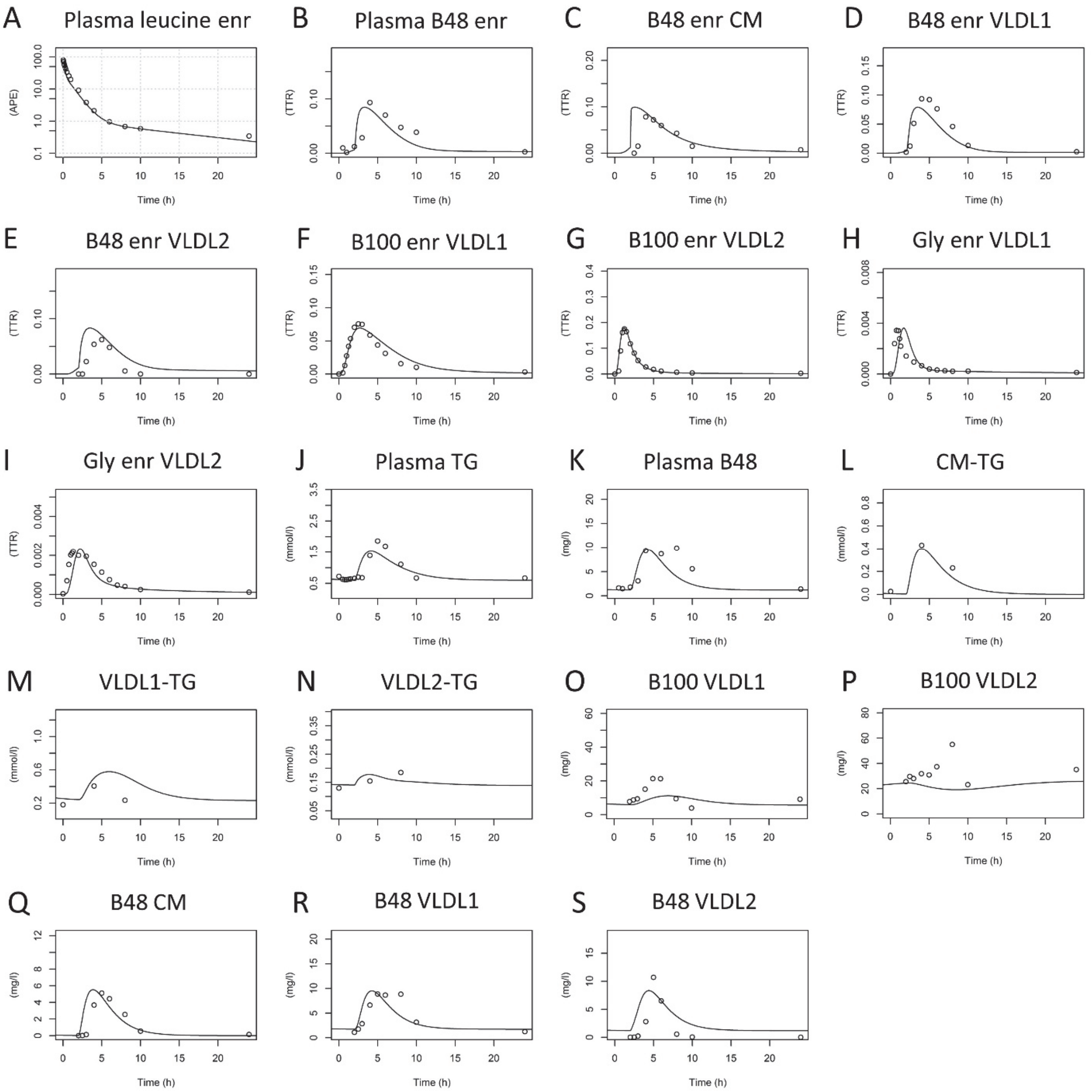

Supplementary figure 4 – subject 1: Model fit to all experimental data for subject 1, 2 and 4 respectively. Plasma leucine enr (enrichment) is plotted in semilogarithmic scale. APE = atom percent excess, TTR = tracer to tracee ratio

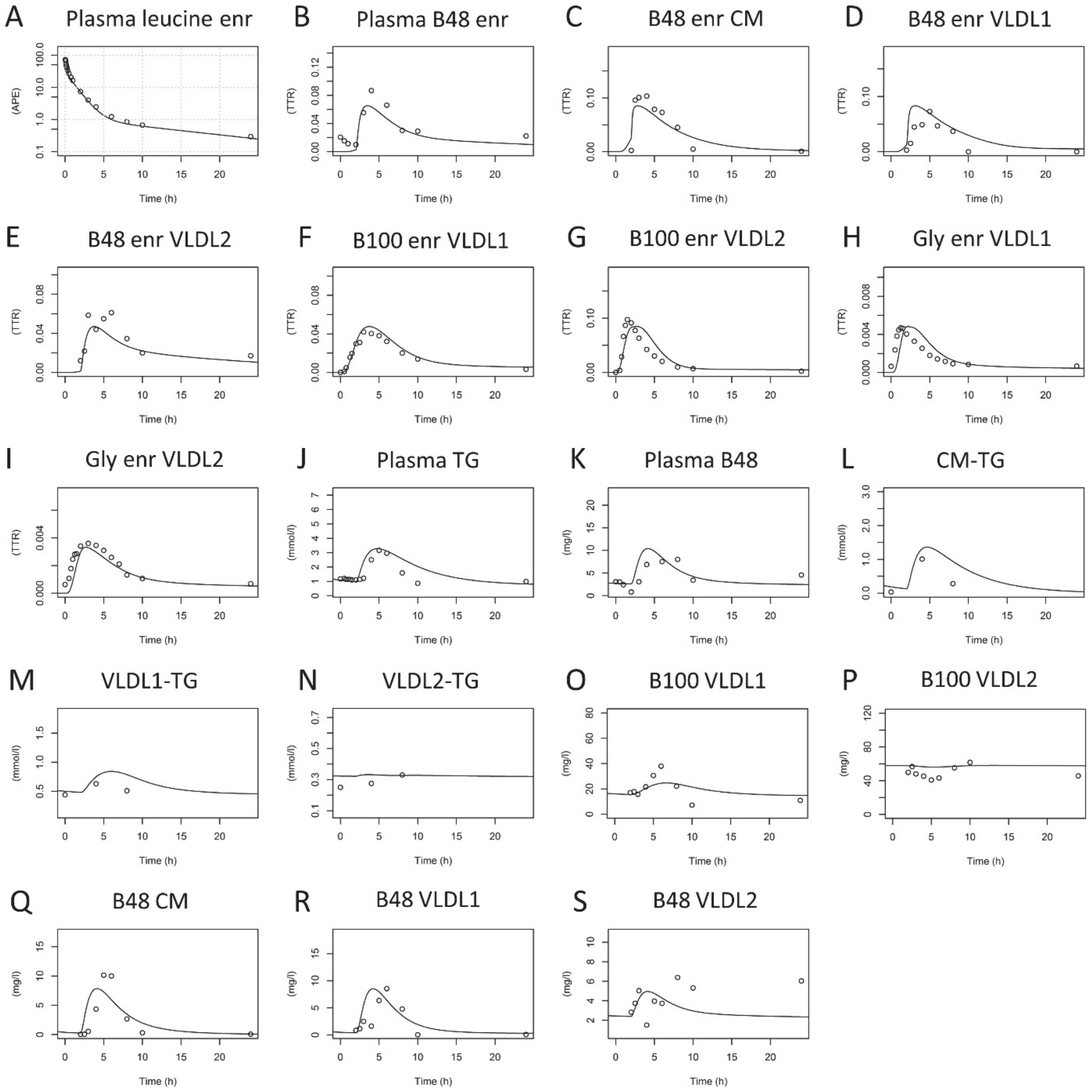

Supplementary figure 4 – subject 2: Model fit to all experimental data for subject 1, 2 and 4 respectively. Plasma leucine enr (enrichment) is plotted in semilogarithmic scale. APE = atom percent excess, TTR = tracer to tracee ratio

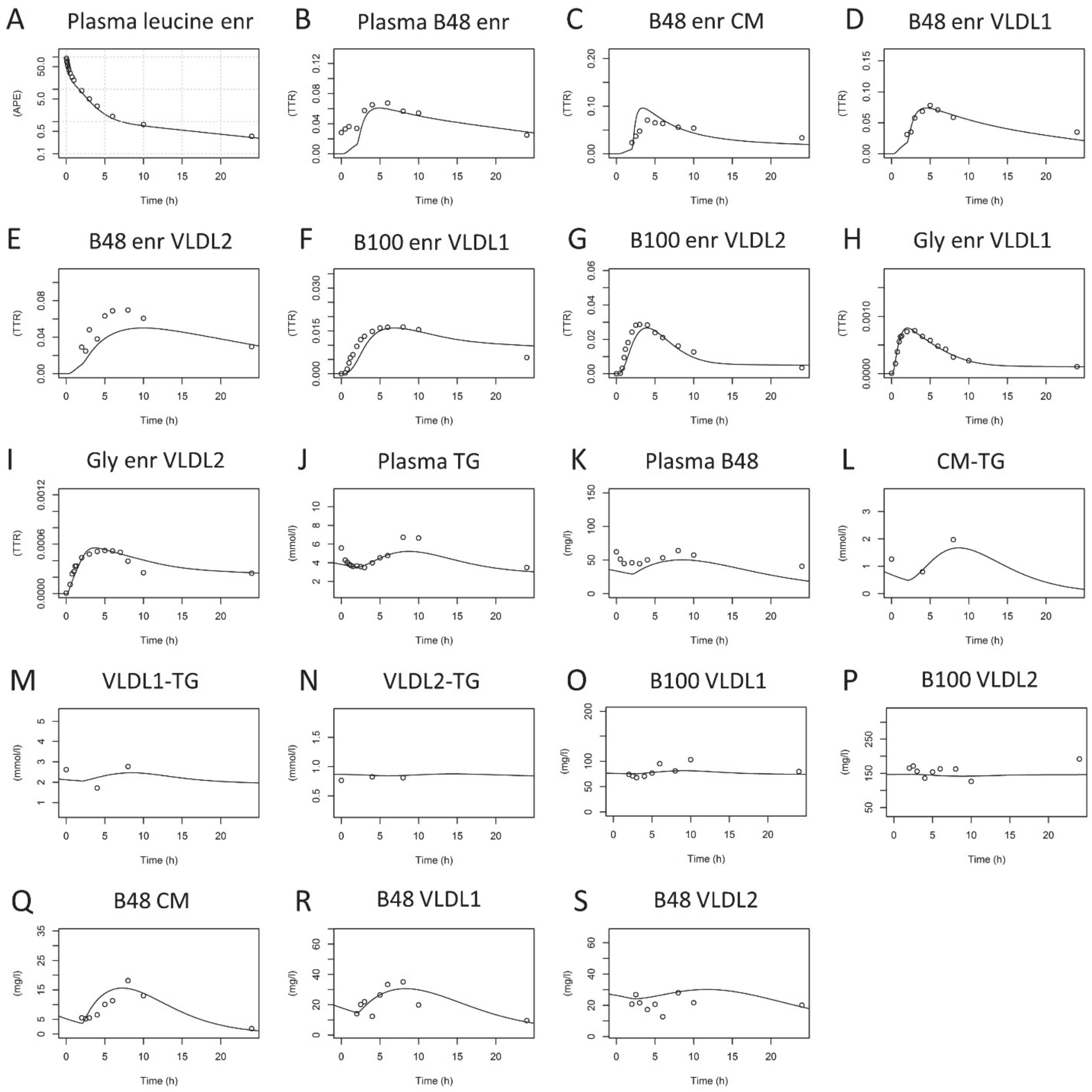

Supplementary figure 4 – subject 4: Model fit to all experimental data for subject 1, 2 and 4 respectively. Plasma leucine enr (enrichment) is plotted in semilogarithmic scale. APE = atom percent excess, TTR = tracer to tracee ratio

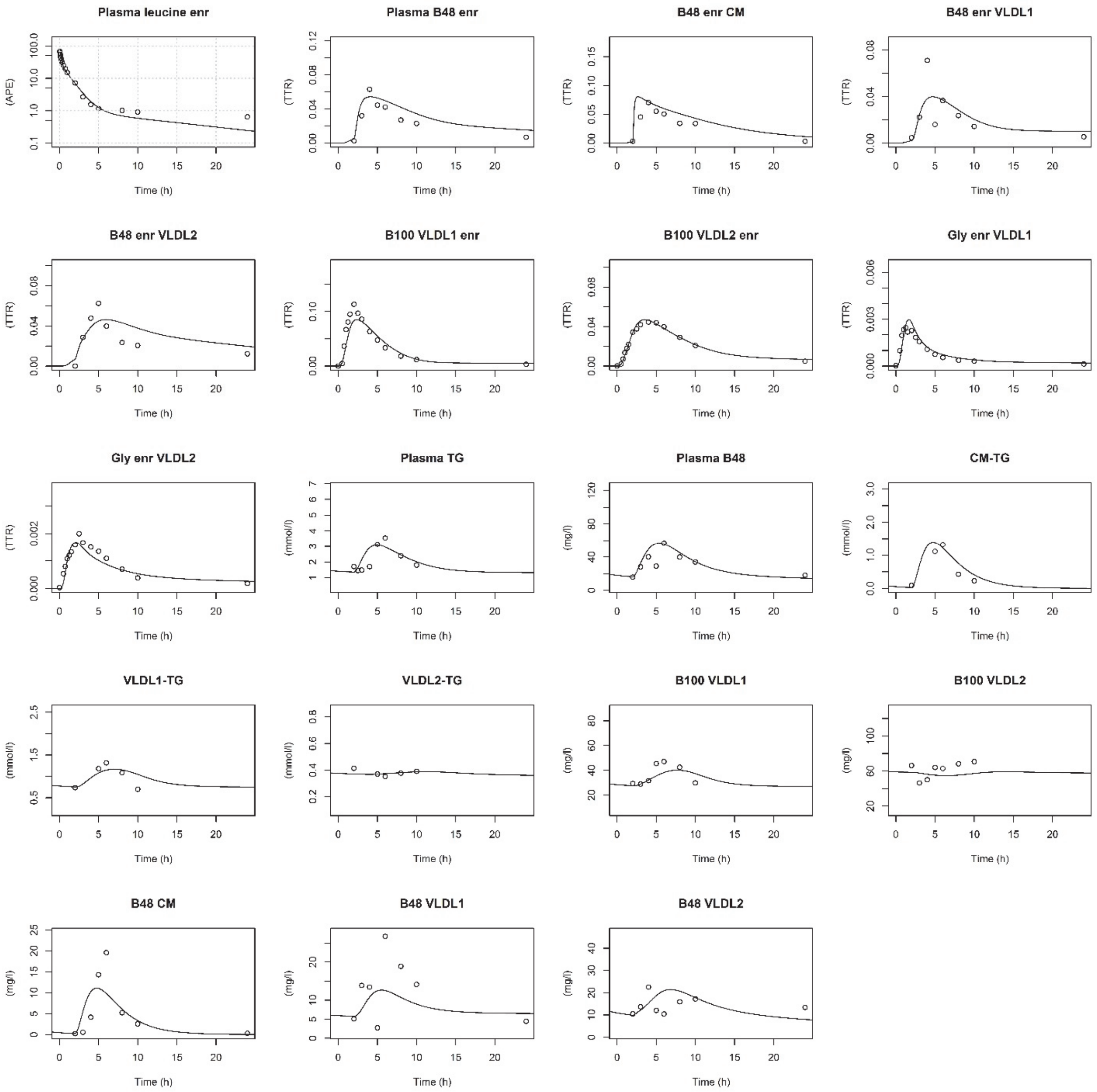

Supplementary Figure 5: subject 5. All model fits to the four subjects in the second cohort are shown below. Generally the model is able to fit the experimental data equally well for the 1st cohort as for the 2nd cohort. Plasma leucine enr (enrichment) is plotted in semilogarithmic scale. APE = atom percent excess, TTR = tracer to tracee ratio

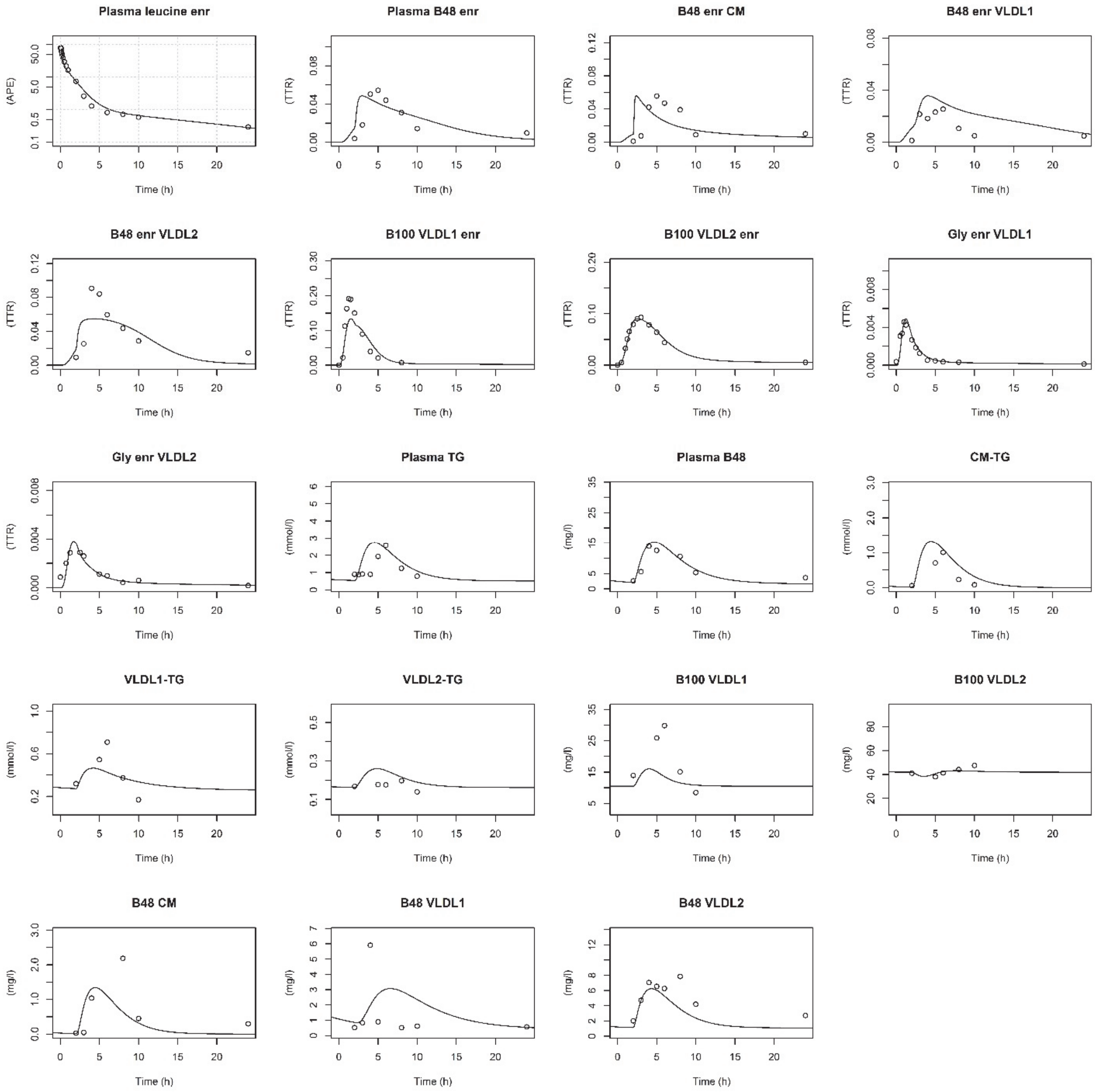

Supplementary Figure 5: subject 6. All model fits to the four subjects in the second cohort are shown below. Generally the model is able to fit the experimental data equally well for the 1st cohort as for the 2nd cohort. Plasma leucine enr (enrichment) is plotted in semilogarithmic scale. APE = atom percent excess, TTR = tracer to tracee ratio

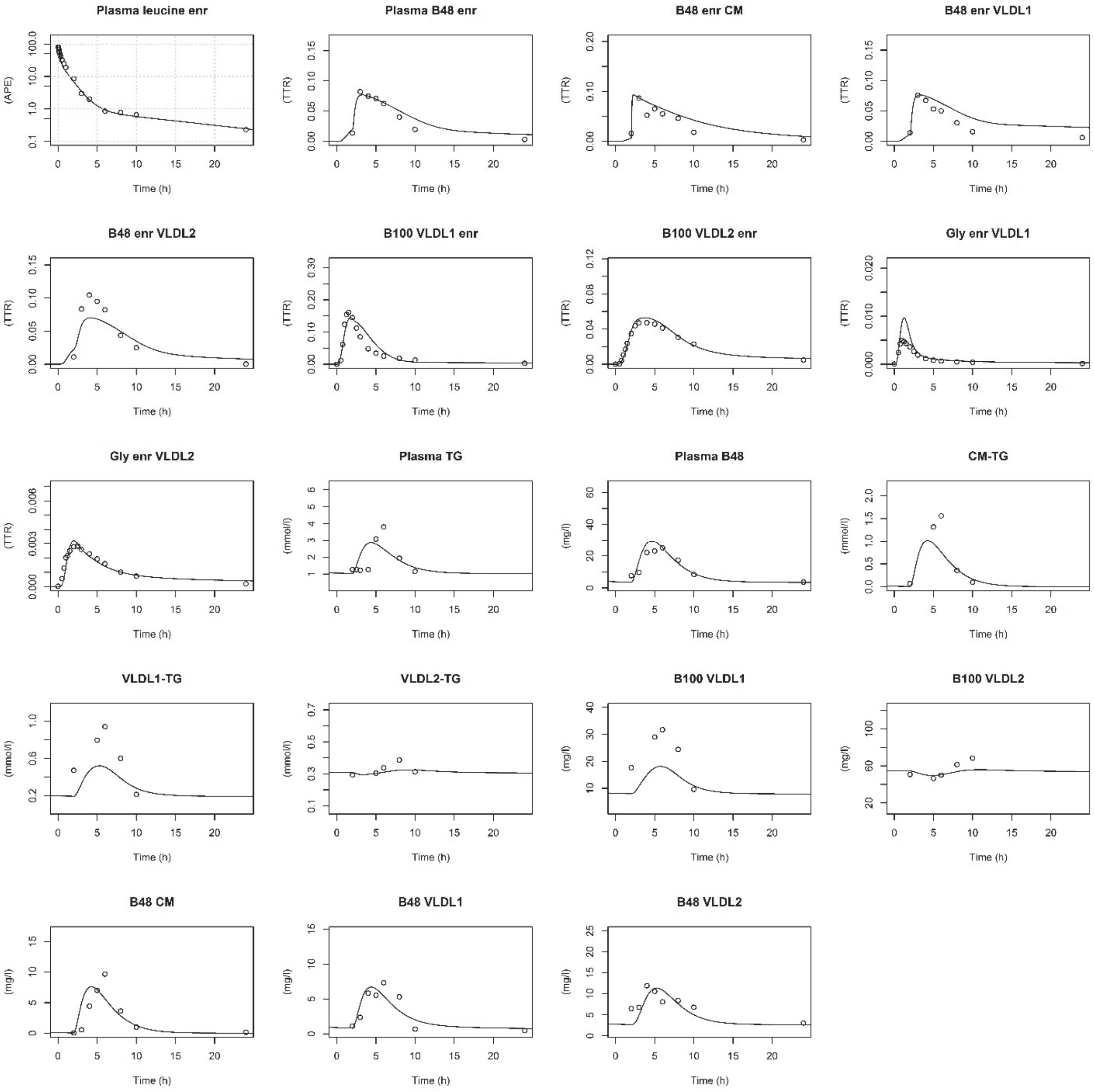

Supplementary Figure 5: subject 7. All model fits to the four subjects in the second cohort are shown below. Generally the model is able to fit the experimental data equally well for the 1st cohort as for the 2nd cohort. Plasma leucine enr (enrichment) is plotted in semilogarithmic scale. APE = atom percent excess, TTR = tracer to tracee ratio

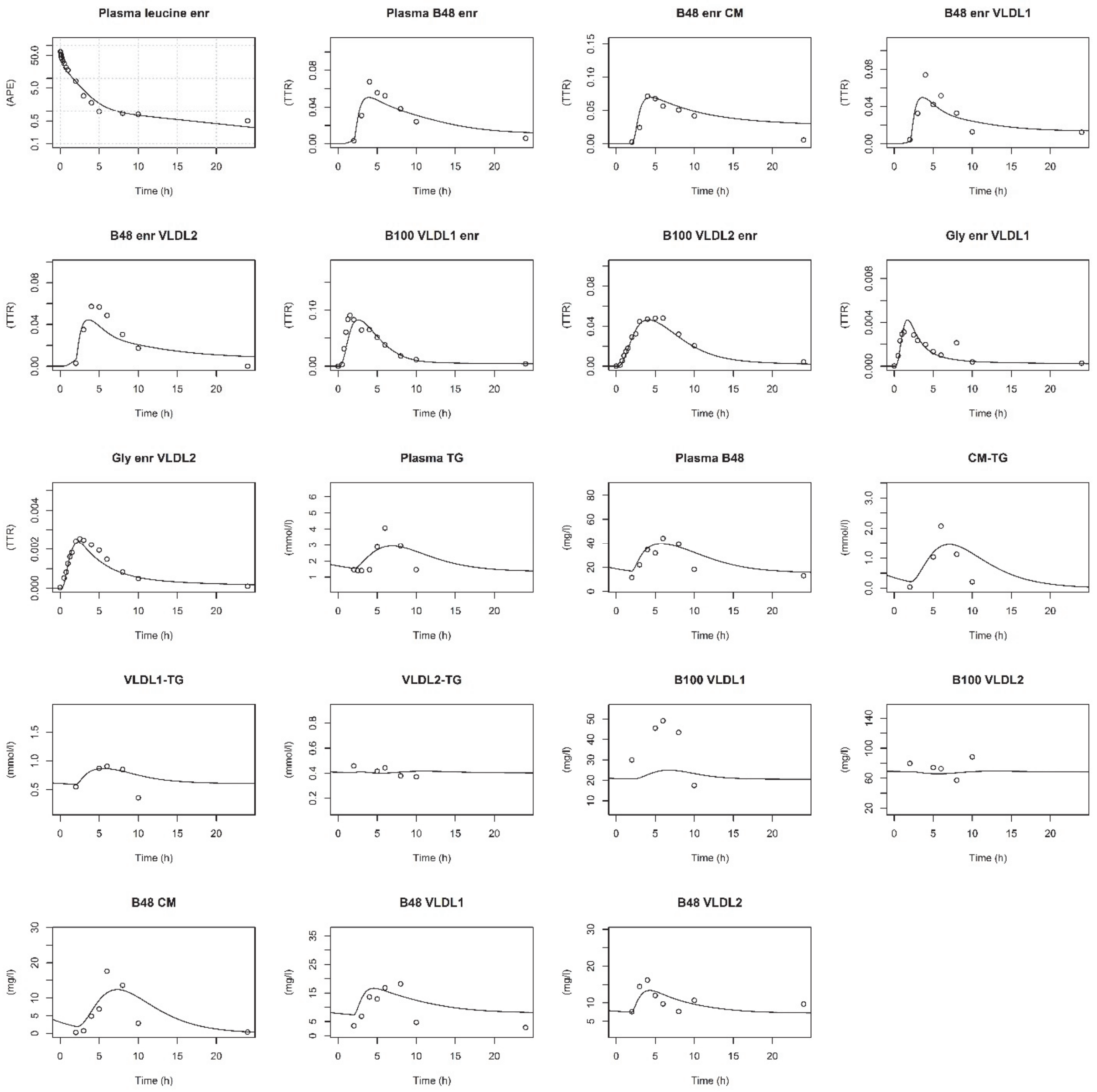

Supplementary Figure 5: subject 8. All model fits to the four subjects in the second cohort are shown below. Generally the model is able to fit the experimental data equally well for the 1st cohort as for the 2nd cohort. Plasma leucine enr (enrichment) is plotted in semilogarithmic scale. APE = atom percent excess, TTR = tracer to tracee ratio

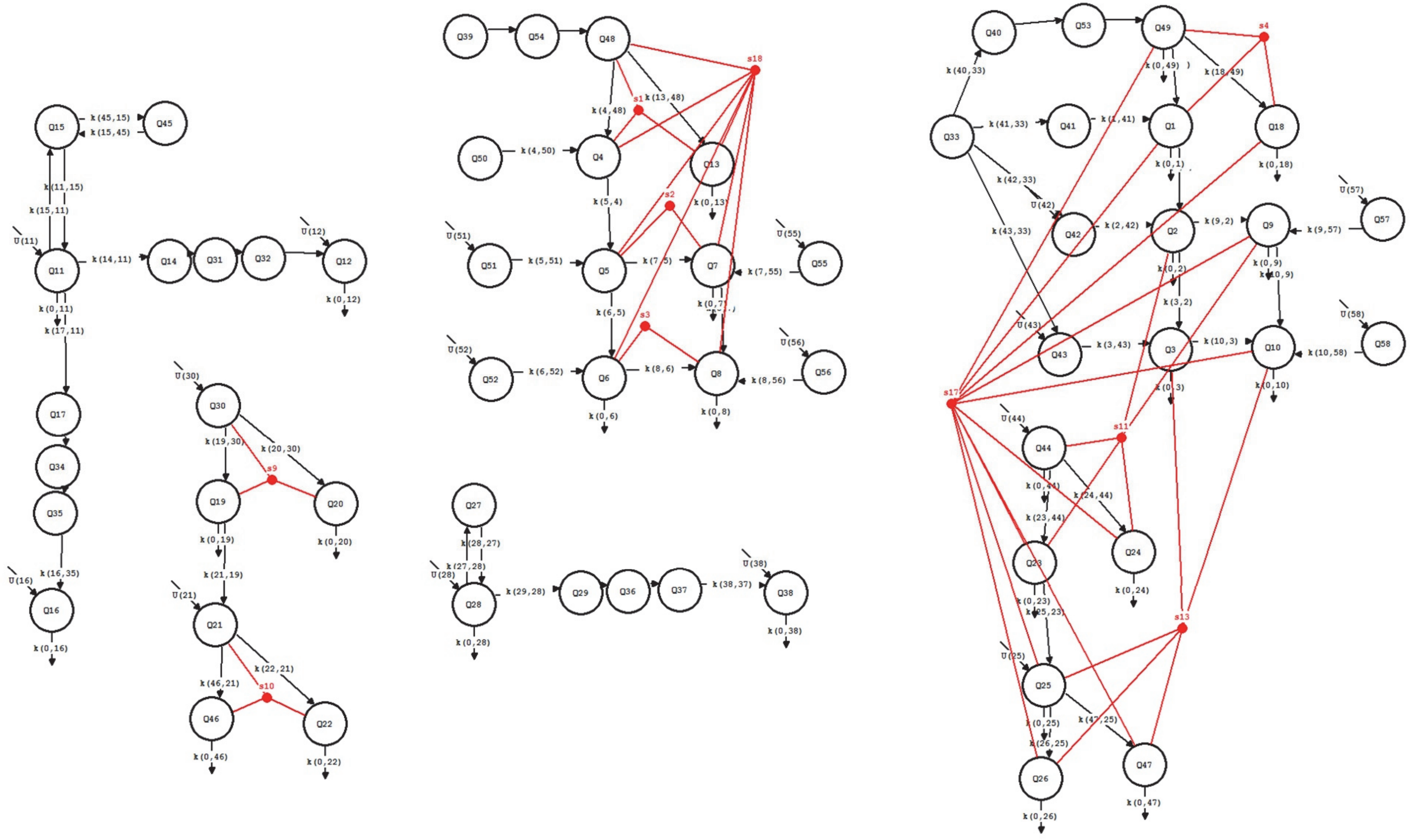

Supplementary Figure 6 — the steady-state level: Full model structure displaying the three "levels" of the model structure (the steady-state level, the tracer level and the non-steady state level). Red lines indicate where experimental data is linked to the model. Blue syringes indicate various "injections" into the model representing either B100/B48 or of tracer

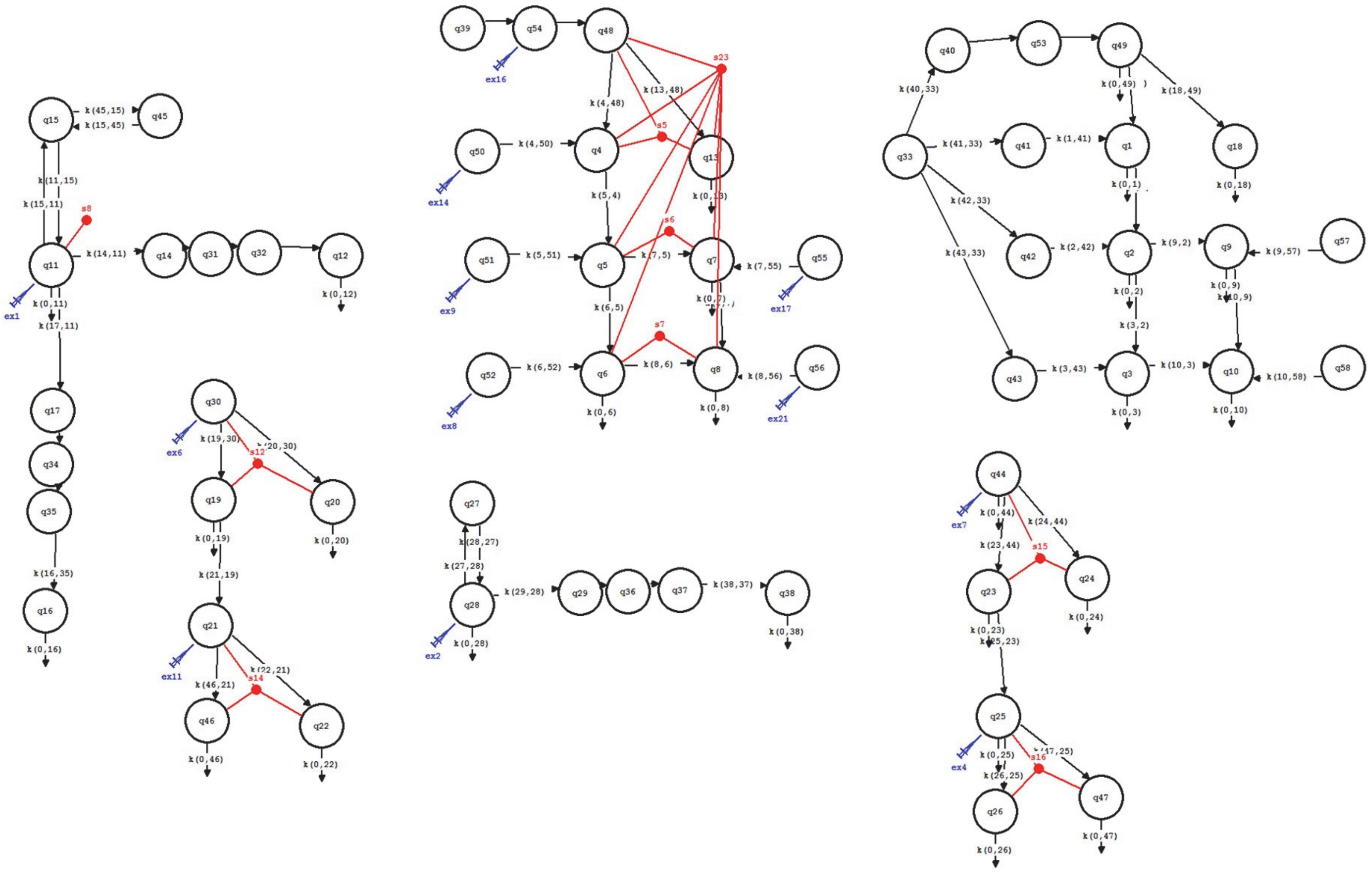

Supplementary Figure 6 — the tracer level: Full model structure displaying the three "levels" of the model structure (the steady-state level, the tracer level and the non-steady state level). Red lines indicate where experimental data is linked to the model. Blue syringes indicate various "injections" into the model representing either B100/B48 or of tracer

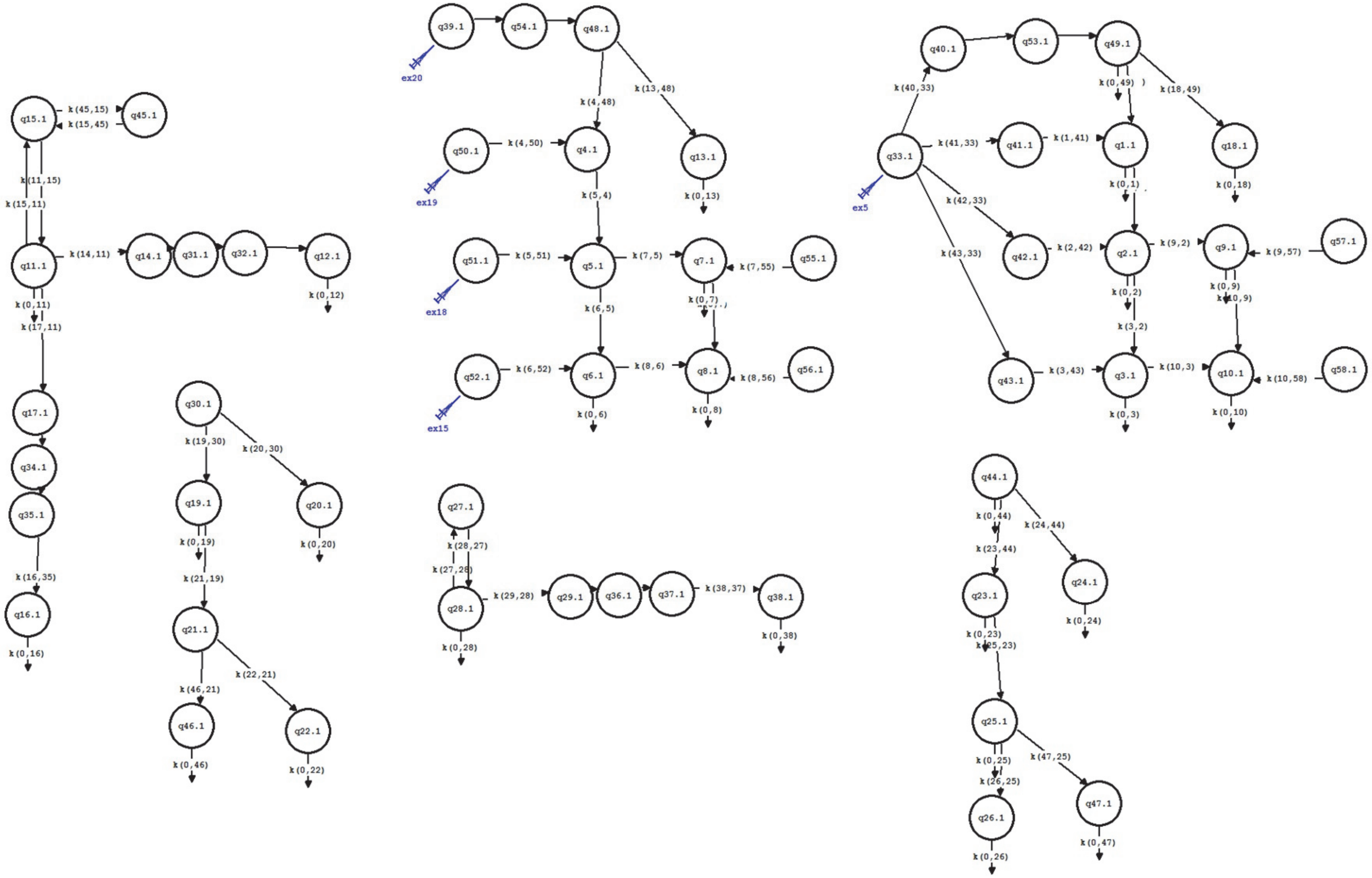

Supplementary Figure 6 — the non-steady state level: Full model structure displaying the three "levels" of the model structure (the steady-state level, the tracer level and the non-steady state level). Red lines indicate where experimental data is linked to the model. Blue syringes indicate various "injections" into the model representing either B100/B48 or of tracer
